# Exploration of bioactive natural products from tropical plant genetic resources: from diversity to functional seeds

**DOI:** 10.1007/s11418-026-02085-8

**Published:** 2026-10-13

**Authors:** Hiroshi Morita, Yusuke Hirasawa, Toshio Kaneda

**Affiliations:** Faculty of Pharmaceutical Sciences, Hoshi University, Ebara 2-4-41, Shinagawa-Ku, Tokyo 142-8501 Japan

**Keywords:** Tropical plant genetic resources, Bioactive natural products, Biodiversity-driven drug discovery, Structure–activity relationship, Mechanism-based pharmacology

## Abstract

Natural products derived from higher plants continue to represent an important source of structurally diverse and biologically active molecules for drug discovery. In particular, tropical plant genetic resources provide a rich reservoir of unique secondary metabolites owing to their exceptional biodiversity. Through long-term collaborative studies conducted mainly in Southeast Asia, extensive collections of tropical plant materials were screened for diverse biological activities, leading to the discovery of numerous bioactive natural products. Early phytochemical studies on medicinal plants such as *Eurycoma longifolia*, a well-known traditional herbal medicine widely used in Malaysia and Indonesia, also contributed to the establishment of our research platform for tropical natural product discovery. This review summarizes representative studies on structurally unique compounds isolated from tropical plant resources and highlights their biological activities and mechanisms of action. Representative examples include ceramicines, limonoids from *Chisocheton ceramicus* with anti-lipid droplet accumulation activity, calofolic acids from *Calophyllum scriblitifolium* exhibiting vasorelaxant effects through PI3K–PKA signaling modulation, cassiarins, alkaloids from *Cassia siamea* with potent antimalarial activity, bisindole alkaloids from *Leuconotis* species inducing autophagy through AKT–mTOR pathway inhibition, cyclolinopeptides from flaxseed suppressing osteoclast differentiation via the c-Fos/RANK axis, celogentins, bicyclic peptides from *Celosia argentea* inhibiting tubulin polymerization, and mollugin derivatives from *Rubia* species targeting TLR4-mediated inflammatory signaling pathways. These studies demonstrate that tropical plant-derived natural products possess remarkable structural diversity and act on biologically relevant molecular targets. Furthermore, several cases illustrate the importance of mechanistic analysis and structural modification for the optimization of pharmacological activity. Collectively, this review highlights the significance of biodiversity-driven natural product exploration as a strategy for identifying functional seeds for future drug discovery.

**Graphical abstract:**

**Figure:**
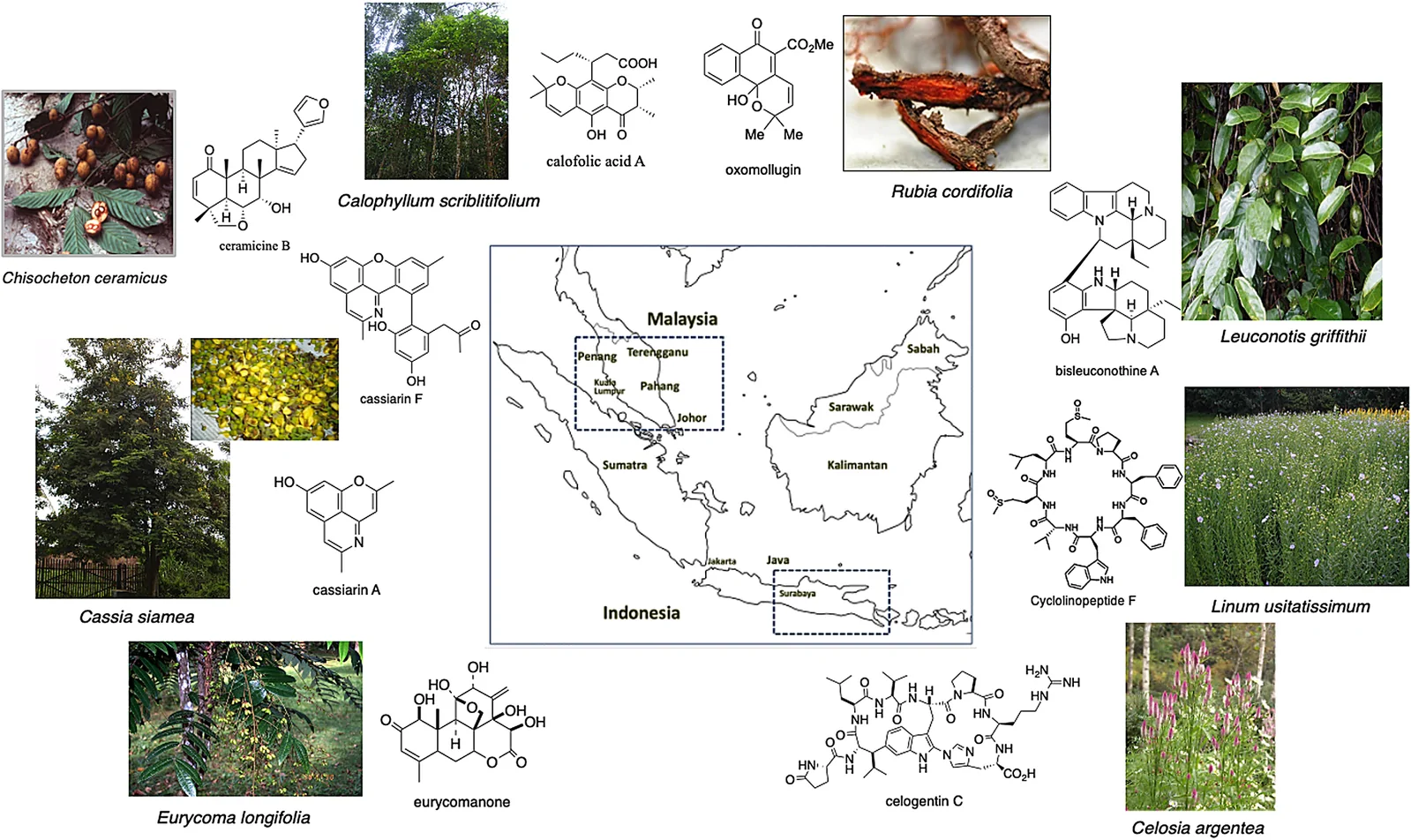

## Introduction

Natural products have long served as an important source of structurally diverse and biologically active molecules for drug discovery. Among natural resources, higher plants represent a particularly rich reservoir of secondary metabolites, many of which remain unexplored. Although more than 300,000 plant species have been identified, only a limited proportion has been investigated from phytochemical and pharmacological perspectives, indicating that plant-derived natural products still constitute a vast and underexplored resource [1–3].

Tropical regions, especially in Southeast Asia, are characterized by exceptional biodiversity and are recognized as major centers of plant genetic resources. Countries such as Indonesia and Malaysia harbor a disproportionately large number of plant species relative to their land area, providing a unique opportunity for the discovery of novel natural products [4–6].

In our research, we have conducted extensive field-based studies in collaboration with local institutions, focusing on tropical plant genetic resources. In Malaysia, collaborative surveys were carried out over several years in partnership with the Faculty of Science, University of Malaya; the Faculty of Science, Universiti Putra Malaysia; and the School of Pharmaceutical Sciences, Universiti Sains Malaysia. These studies involved systematic exploration of forest areas in regions such as Terengganu, Pahang, Johor, Penang, Cameron Highlands, Mersing, and Kuantan.

Similarly, in Indonesia, long-term collaborative investigations were conducted primarily in East Java in cooperation with the Faculty of Pharmacy, Universitas Airlangga, and the Purwodadi Botanic Garden. These joint efforts enabled the collection of a large number of plant materials from diverse ecological environments.

Through the collection and screening of these plant materials, we have established a platform for the discovery of bioactive natural products. This approach integrates bioactivity-guided isolation, structure elucidation, mechanistic studies, and, when necessary, chemical synthesis and derivatization [7, 8].

Among the medicinal plants investigated during these collaborative studies, *Eurycoma longifolia* Jack (Simaroubaceae), commonly known as Tongkat Ali, represents one of the most important traditional herbal medicines in Malaysia and Indonesia. Our early phytochemical investigations of this species led to the isolation of structurally unique quassinoids, squalene-type triterpenes, and neolignans, demonstrating the remarkable chemical diversity of tropical medicinal plants and providing an important foundation for subsequent natural product exploration studies [9–21].

**Fig. 1:**
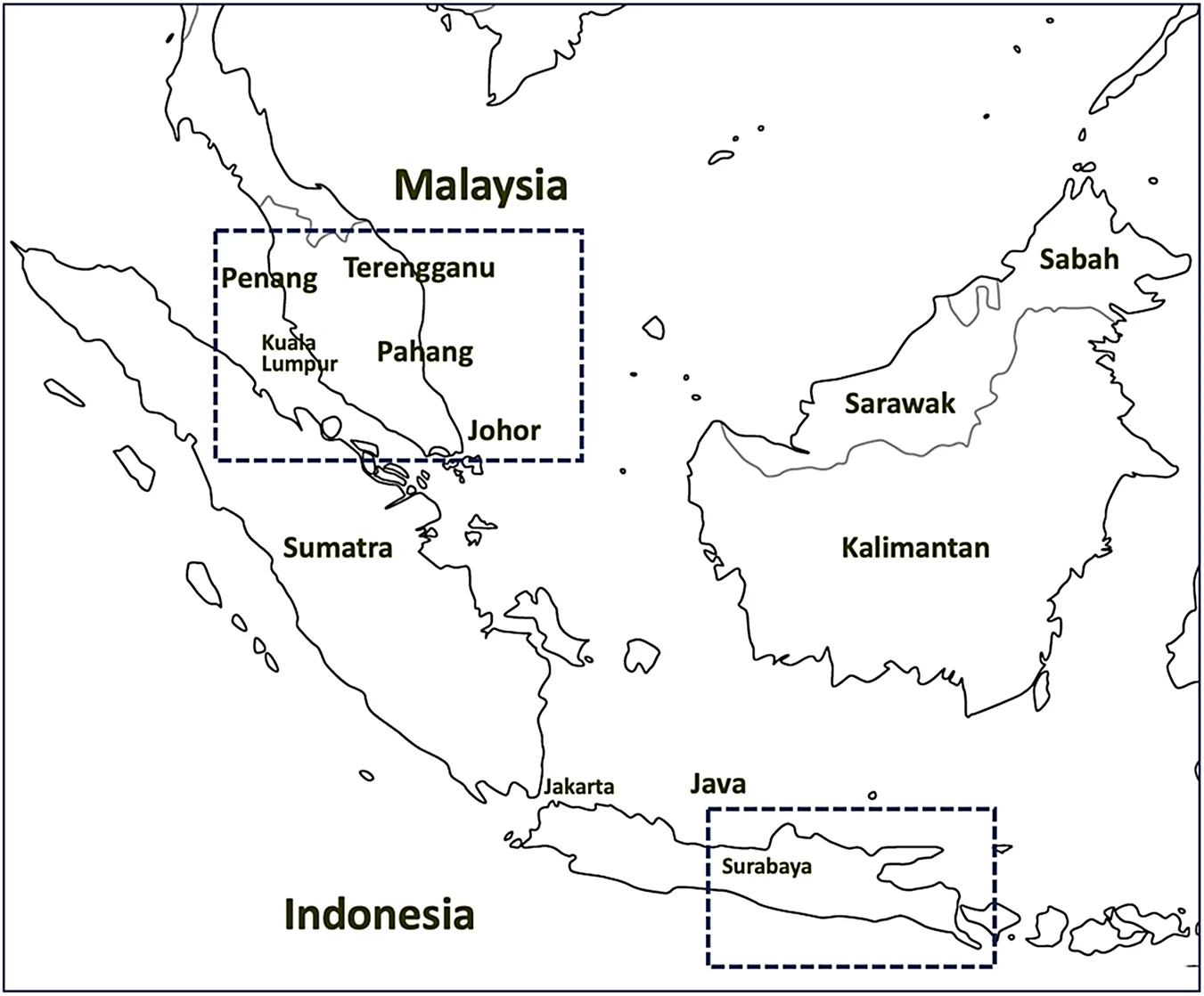
Geographic locations of field surveys conducted in Malaysia and Indonesia

These early investigations, together with subsequent collaborative studies on diverse tropical plant resources, established a research framework integrating biodiversity exploration, natural product chemistry, and mechanism-oriented pharmacological evaluation. In this review, rather than providing an exhaustive list, we highlight seven representative case studies that illustrate the potential of tropical plant-derived natural products as functional seeds for drug discovery. These examples include compounds exhibiting anti-lipid droplet accumulation, vasorelaxant, antimalarial, autophagy-inducing, osteoclast differentiation inhibitory, tubulin polymerization inhibitory, and anti-inflammatory activities (see Fig. 1).

## Exploration of tropical plant genetic resources

Tropical regions, particularly in Southeast Asia, represent one of the most important reservoirs of plant genetic resources, characterized by exceptional biodiversity and a high degree of endemism. Countries such as Indonesia and Malaysia are classified as mega-diversity regions, where a remarkably large number of plant species are concentrated within relatively limited geographical areas. These environments, shaped by complex ecological interactions and evolutionary pressures, are expected to promote the production of structurally diverse and biologically active secondary metabolites [4, 5, 22].

In our research, extensive field investigations have been conducted in collaboration with local universities and research institutions in these regions. These collaborative frameworks have enabled the systematic collection and identification of a wide range of plant materials, including underexplored species, endemic plants, and those traditionally used in local medicinal practices. In particular, ethnopharmacological information has often provided useful guidance for selecting candidate species, although non-traditional and taxonomically unique plants have also been included to avoid bias in sample selection [23, 24].

Plant materials collected in the field were processed to generate crude extracts using appropriate solvents, and a large number of extracts were prepared for subsequent biological evaluation. These extracts were subjected to screening using multiple assay systems designed to detect diverse biological activities, such as modulation of lipid metabolism, vascular function, cell differentiation, cytoskeletal dynamics, and inflammatory signaling pathways. As a result, numerous extracts exhibiting significant bioactivities were identified, providing a rich source for further chemical investigation [7, 25].

A key feature of this research is the scale and diversity of the screened materials. Rather than focusing on a limited number of well-studied species, our approach involves the systematic evaluation of a broad collection of plant extracts obtained through long-term collaborative efforts. This strategy increases the likelihood of identifying structurally novel compounds and reduces redundancy in natural product discovery. Importantly, the representative compounds highlighted in this review were selected from this large pool of screened samples, emphasizing that they reflect the outcomes of extensive exploration rather than isolated findings [1, 7].

Another important aspect of this work is the establishment of sustainable and ethical research practices. All field studies and sample collections have been conducted in close collaboration with local partners and in compliance with national regulations and international frameworks, including the Convention on Biological Diversity and Access and Benefit-Sharing agreements. These considerations are essential for ensuring the responsible utilization of biological resources and for maintaining long-term research partnerships [26, 27].

Through the integration of biodiversity-driven sample selection, large-scale extract screening, and international collaboration, this research framework provides a robust foundation for the discovery of bioactive natural products. The following sections describe selected case studies that exemplify the outcomes of this approach.

## Strategy for bioactive natural product discovery

The discovery of bioactive natural products in this study is based on a standard workflow combining biological and chemical approaches. Active plant extracts identified through screening are subjected to bioactivity-guided fractionation, leading to the isolation of individual compounds. Structural elucidation is performed using spectroscopic techniques such as NMR and mass spectrometry, and, when necessary, X-ray crystallography.

The biological activities of isolated compounds are further evaluated, and their mechanisms of action are investigated at the molecular level. In selected cases, chemical synthesis or structural modification is conducted to confirm structures and to examine structure–activity relationships.

## Limonoids from *Chisocheton ceramicus*: from multifunctional activities to lipid droplet accumulation inhibition

**Fig. 2:**
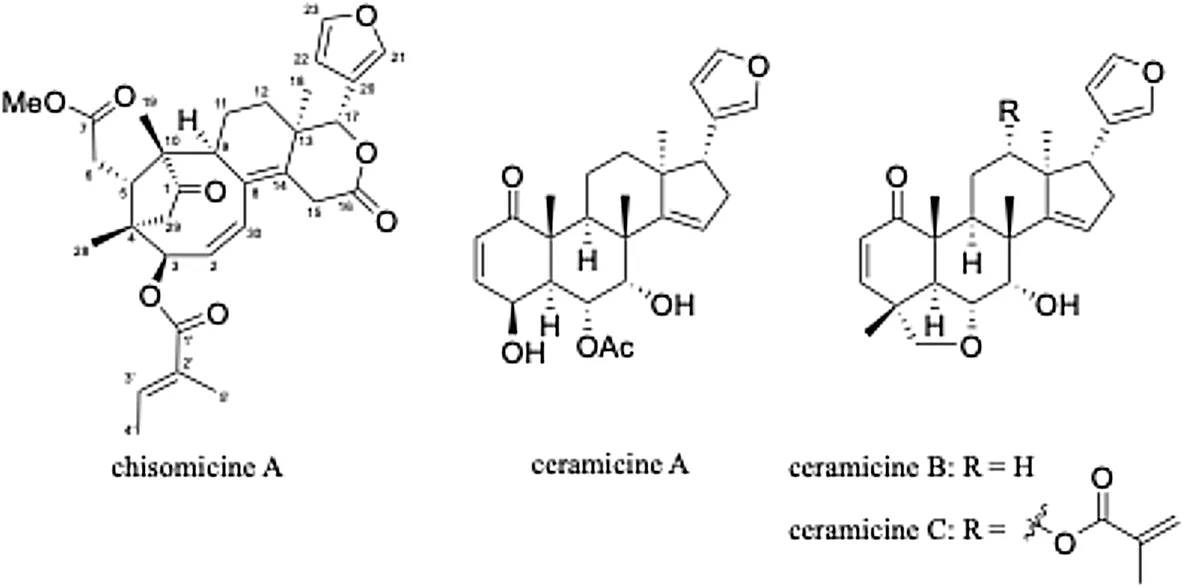
Structures of chisomicine A and ceramicines A-C from *Chisocheton ceramicus*

Limonoids isolated from *Chisocheton ceramicus* (Meliaceae), collectively known as ceramicines, represent a structurally unique class of highly oxidized triterpenoids characterized by a polycyclic framework with diverse oxygenated functionalities. Since the initial isolation of chisomicines [28, 29] and ceramicines A–C [30, 31], a large number of analogues, currently extending from ceramicines A to Z, have been identified through continuous phytochemical investigations of this plant [30–40] (see Fig. 2).

**Fig. 3:**
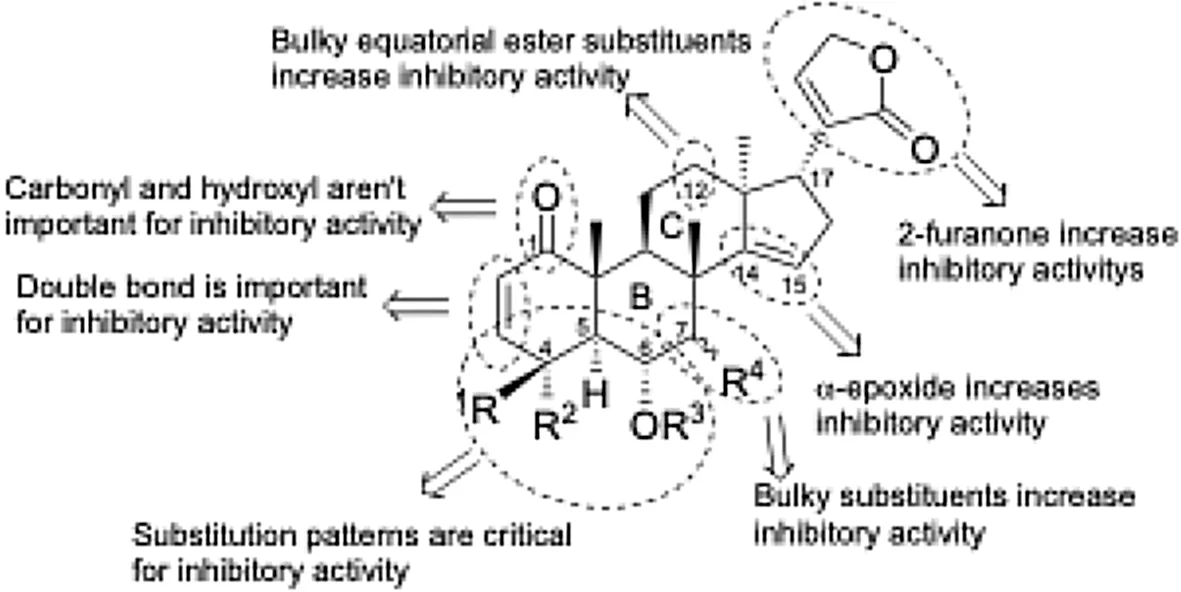
SAR of ceramicines for antimalarial activity

Early studies revealed that ceramicines exhibit a wide range of biological activities. For example, ceramicine A showed moderate cytotoxic activity against murine leukemia P388 cells [30], while ceramicine B exhibited potent antimalarial activity against *Plasmodium falciparum* 3D7 strain [31]. Subsequent studies further expanded the biological profile of this compound class, including anti-melanin deposition activity in melanoma cells [36], where ceramicine B was shown to reduce tyrosinase protein expression without affecting its enzymatic activity. In addition, continuous isolation efforts led to the discovery of new analogues, including ceramicines Q–T and U–Z, some of which retained strong antimalarial activity with micromolar potency [39, 40] (see Fig. 3).

These findings indicate that ceramicines possess multifunctional biological properties, reflecting their structurally diverse and highly functionalized frameworks. However, despite this diversity of biological activities, a particularly notable feature of ceramicines is their ability to modulate lipid metabolism [34, 35].

During our screening for inhibitors of lipid droplet accumulation (LDA), the extract of *C. ceramicus* was found to exhibit significant inhibitory activity in a mouse preadipocyte cell line (MC3T3-G2/PA6). Bioactivity-guided fractionation led to the identification of ceramicine B as a major active component. Further evaluation demonstrated that ceramicine B suppresses lipid droplet accumulation in a dose-dependent manner, indicating its potential as an anti-adipogenic agent [34].

Mechanistic studies revealed that ceramicine B downregulates the expression of adipogenesis-related genes, including glucose transporter type 4 (GLUT4), lipoprotein lipase (LPL), and 11β-hydroxysteroid dehydrogenase. In addition, the expression of key transcription factors involved in adipocyte differentiation, such as peroxisome proliferator-activated receptor γ (PPARγ) and CCAAT/enhancer-binding protein α (C/EBPα), was significantly suppressed. Notably, early adipogenic regulators, including C/EBPβ and C/EBPδ, were not affected, suggesting that ceramicine B acts at a relatively late stage of adipocyte differentiation [35].

**Fig. 4:**
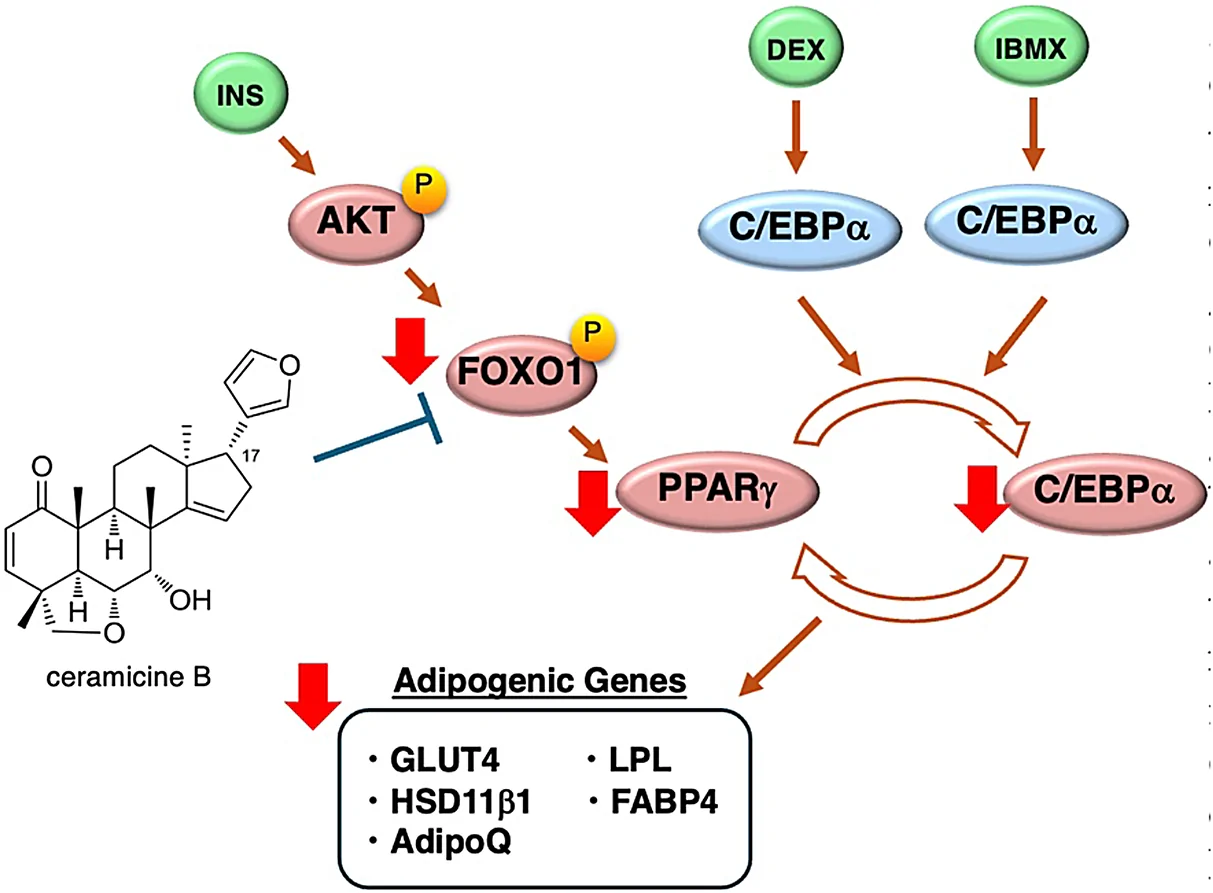
Proposed mechanism of action for the inhibitory effect of ceramicine B on lipid droplet accumulation. Ceramicine B was identified as an active compound that inhibits lipid droplet accumulation (LDA) in the mouse preadipocyte cell line MC3T3-G2/PA6. Ceramicine B suppressed the mRNA expression of glucose transporter 4 (GLUT4), lipoprotein lipase (LPL), and 11β-hydroxysteroid dehydrogenase (11β-HSD), as well as that of peroxisome proliferator-activated receptor γ (PPARγ) and CCAAT/enhancer-binding protein α (C/EBPα)—master regulators of adipocyte differentiation. In contrast, it did not affect the expression of early adipocyte differentiation regulators such as C/EBPβ and C/EBPδ, or the intermediate-stage regulator Krüppel-like factor (KLF). Western blot analysis revealed that ceramicine B inhibited the phosphorylation of Forkhead box O1 (Foxo1), a key process in the insulin signaling pathway. These findings suggest that the inhibition of Foxo1 phosphorylation is partially involved in the mechanism underlying the anti-adipogenic effect of ceramicine B

At the signaling level, ceramicine B was found to inhibit the phosphorylation of Forkhead box O1 (FoxO1), a key regulator in the insulin signaling pathway. This observation suggests that the anti-LDA activity of ceramicine B is mediated, at least in part, through modulation of the AKT–FoxO1 signaling axis [35] (see Fig. 4).

Structure–activity relationship (SAR) studies further supported the importance of specific structural features for LDA inhibitory activity. Analysis of ceramicines A–L and newly identified analogues indicated that substituents around the B and C rings, particularly at positions such as C-7, significantly influence activity and selectivity. These findings suggest that subtle modifications in the oxygenation pattern and stereochemistry of the limonoid skeleton can markedly alter biological activity [37].

**Fig. 5:**
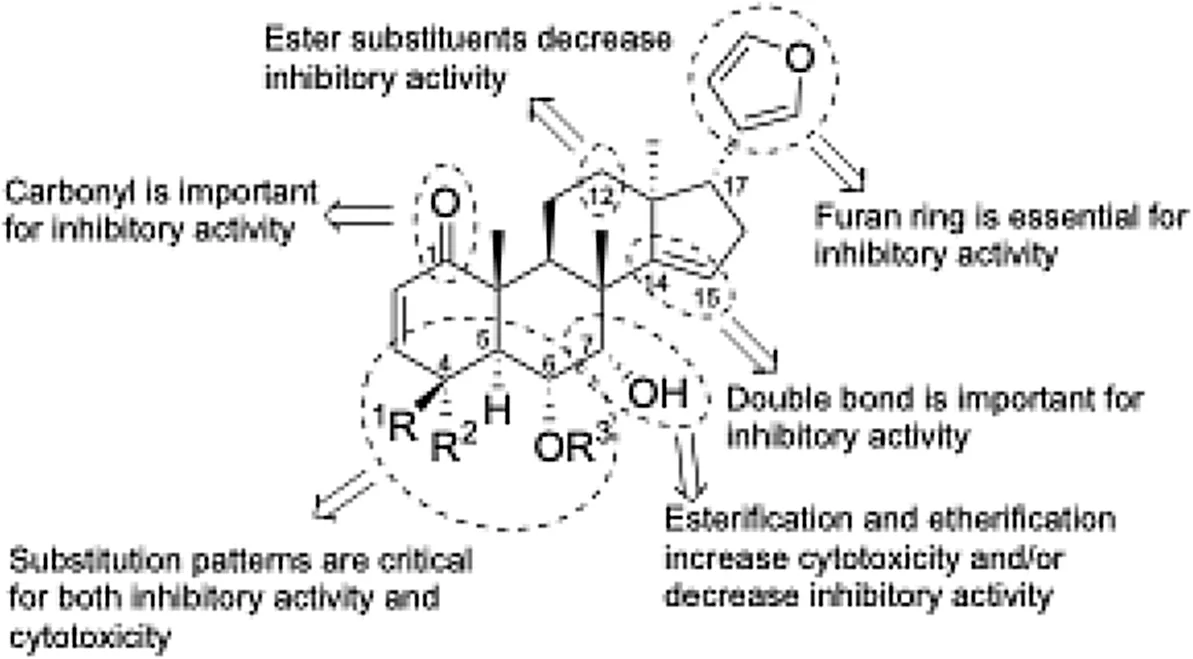
Summarized SAR of ceramicines as LDA inhibitors

Taken together, although ceramicines exhibit a broad spectrum of biological activities, their anti-lipid droplet accumulation activity represents a particularly promising pharmacological property. The combination of a unique limonoid scaffold, tunable structural features, and a defined molecular mechanism highlights ceramicines as attractive candidates for the development of anti-obesity agents targeting adipocyte differentiation and lipid metabolism (see Fig. 5).

## Chromanones from *Calophyllum scriblitifolium*: vasorelaxant activity of calofolic acid A

*Calophyllum scriblitifolium* (Calophyllaceae) is a tropical tree distributed in Malaysia and Indonesia. The genus *Calophyllum* is known to produce diverse secondary metabolites, including xanthones, coumarins, acylphloroglucinols, terpenoids, and chromanones. In the course of our screening of tropical plant extracts for vasorelaxant activity, the methanol extract of the bark of *C. scriblitifolium* was found to relax phenylephrine-precontracted rat aortic rings [41].

Bioactivity-guided separation of the active extract led to the isolation of six chromanones, calofolic acids A–F. Their structures were elucidated by 1D and 2D NMR spectroscopy, and their absolute configurations were investigated using CD spectroscopy in combination with DFT calculations. All isolated calofolic acids showed dose-dependent vasorelaxant activity in isolated rat aorta, indicating that the chromanone scaffold contributes to this activity [41].

**Fig. 6:**
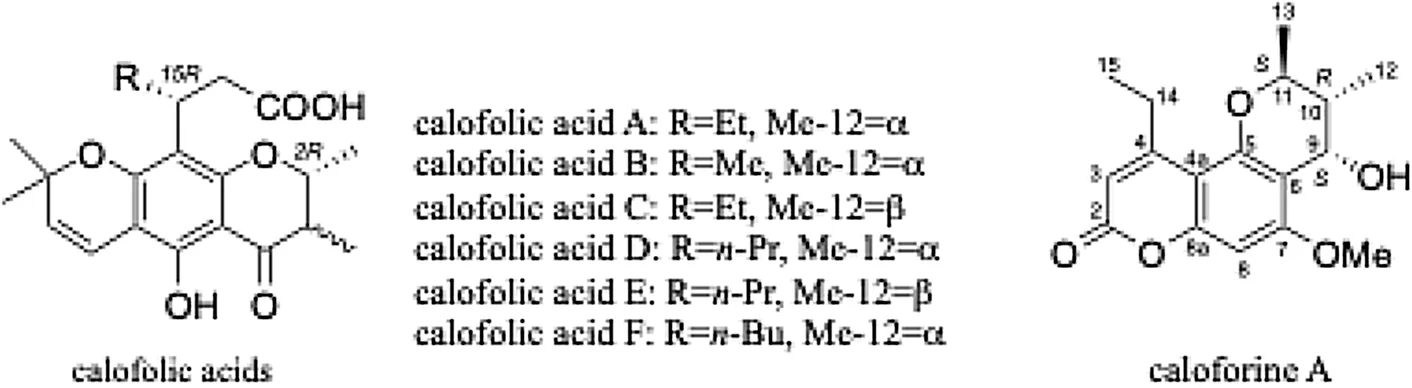
Structures of calofolic acids and caloforine A from *Calophyllum scriblitifolium*

Further phytochemical investigation of the same plant also afforded structurally distinct coumarins, caloforines A–G, which exhibited moderate antimalarial activity against *Plasmodium falciparum* 3D7 strain [42]. These findings indicate that *C. scriblitifolium* produces multiple classes of bioactive compounds, including chromanones and coumarins, with different pharmacological profiles. Among them, however, calofolic acid A was selected for detailed pharmacological analysis because it was a major constituent of the active fraction and exhibited clear vasorelaxant activity (see Fig. 6).

**Fig. 7:**
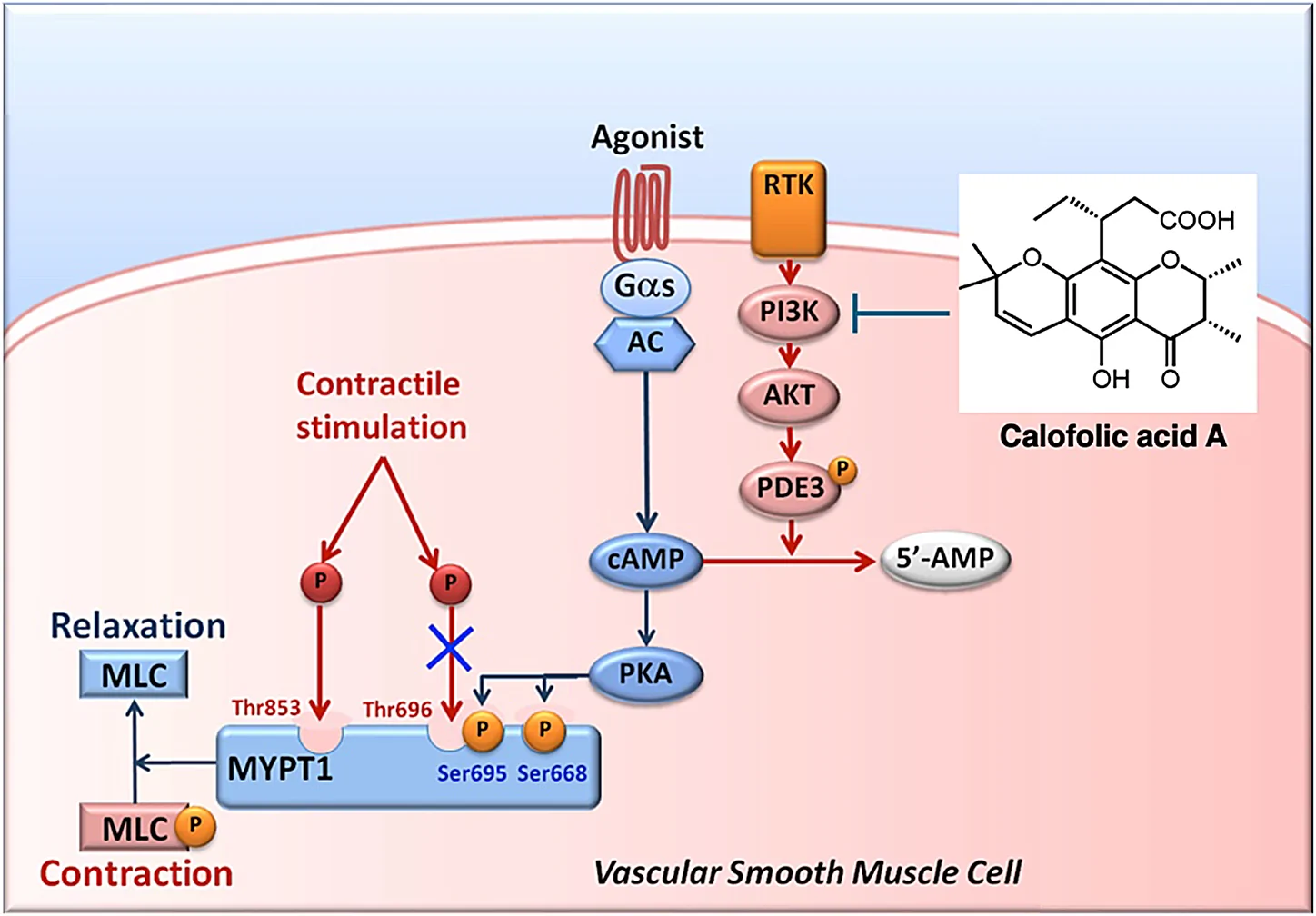
Proposed mechanism for the vasorelaxant effect of calofolic acid A in VSMCs. The detailed mechanism underlying the vasorelaxant effect of calofolic acid A (CA-A) was investigated using rat vascular smooth muscle cells (VSMCs) and aortic ring preparations. In VSMCs pretreated with CA-A, phenylephrine (PE)-induced phosphorylation of MLC at Ser19 was reduced. CA-A also decreased PE-induced phosphorylation of MYPT1 at Thr696 and Thr853. Conversely, CA-A increased PE-induced phosphorylation of MYPT1 at Ser695 and Ser668, sites known to be phosphorylated by cAMP-dependent protein kinase (PKA). CA-A slightly enhanced PKA substrate phosphorylation in a concentration-dependent manner and further potentiated isoproterenol (ISO)-induced cAMP accumulation and PKA substrate phosphorylation. Treatment with the PI3-kinase (PI3K) inhibitor LY294002 also enhanced ISO-induced cAMP accumulation and PKA substrate phosphorylation, similar to the effects of CA-A. Additionally, CA-A was found to directly inhibit PI3K enzymatic activity in a dose-dependent manner. These findings suggest that CA-A induces vasorelaxation by indirectly activating the PKA-MYPT1 pathway through the inhibition of PI3K activity

Mechanistic studies using rat vascular smooth muscle cells demonstrated that calofolic acid A suppresses phenylephrine-induced phosphorylation of myosin light chain at Ser19, a key event in smooth muscle contraction. In addition, calofolic acid A decreased phosphorylation of MYPT1 at Thr696 and Thr853, which are inhibitory phosphorylation sites, while increasing phosphorylation of MYPT1 at Ser695 and Ser668, sites associated with relaxation signaling. These results suggest that calofolic acid A promotes myosin light-chain phosphatase activity and thereby reduces MLC phosphorylation [43] (see Fig. 7).

Calofolic acid A also enhanced PKA substrate phosphorylation and increased isoproterenol-induced cAMP accumulation. Its effects resembled those of the PI3K inhibitor LY294002, and calofolic acid A was shown to directly inhibit PI3K enzyme activity in a dose-dependent manner. These findings indicate that calofolic acid A induces vasorelaxation through indirect activation of the cAMP–PKA pathway caused by inhibition of PI3K activity [43].

Overall, the study of *C. scriblitifolium* illustrates how screening of tropical plant extracts can lead not only to the discovery of structurally new compounds but also to the identification of biologically meaningful mechanisms. Although this plant yielded several bioactive constituents, including antimalarial caloforines, the calofolic acids are particularly notable as chromanone-type vasorelaxant compounds. Among them, calofolic acid A provides a representative example of a tropical plant-derived natural product that modulates vascular smooth muscle contraction through the PI3K–cAMP–PKA–MYPT1–MLC signaling pathway [43].

## Antimalarial alkaloids from *Cassia siamea*: structural diversity, chemical elaboration, and convergence to antimalarial activity

*Cassia siamea* (Leguminosae) is widely distributed in Southeast Asia and has long been used in traditional medicine, particularly for the treatment of periodic fever and malaria. Phytochemical investigation of this plant has led to the discovery of a unique family of alkaloids and related compounds with structurally diverse frameworks and multiple biological activities, ultimately converging on potent antimalarial effects.

**Discovery of cassiarins: a novel alkaloid scaffold**. The initial breakthrough was the isolation of cassiarins A and B, which possess an unprecedented tricyclic aromatic alkaloid skeleton [44]. Cassiarin A exhibited potent antiplasmodial activity against *Plasmodium falciparum* in vitro and also showed in vivo efficacy against *P. berghei*, highlighting its potential as a lead compound for antimalarial drug development [47]. The identification of this novel scaffold represents a notable example of how traditional medicinal plants can yield structurally unique and pharmacologically relevant natural products.

**Expansion of structural diversity**. Subsequent investigations significantly expanded the structural diversity of this alkaloid family. Cassiarins C–E were isolated as additional analogues, including dimeric structures such as cassiarins D and E, which consist of linked chromone and cassiarin units [47]. Further studies led to the isolation of cassiarins G, H, J, and K, which exhibit variations in oxidation patterns and substitution, and whose structures were rigorously established by spectroscopic analysis and, in some cases, X-ray crystallography [50].

**Fig. 8:**
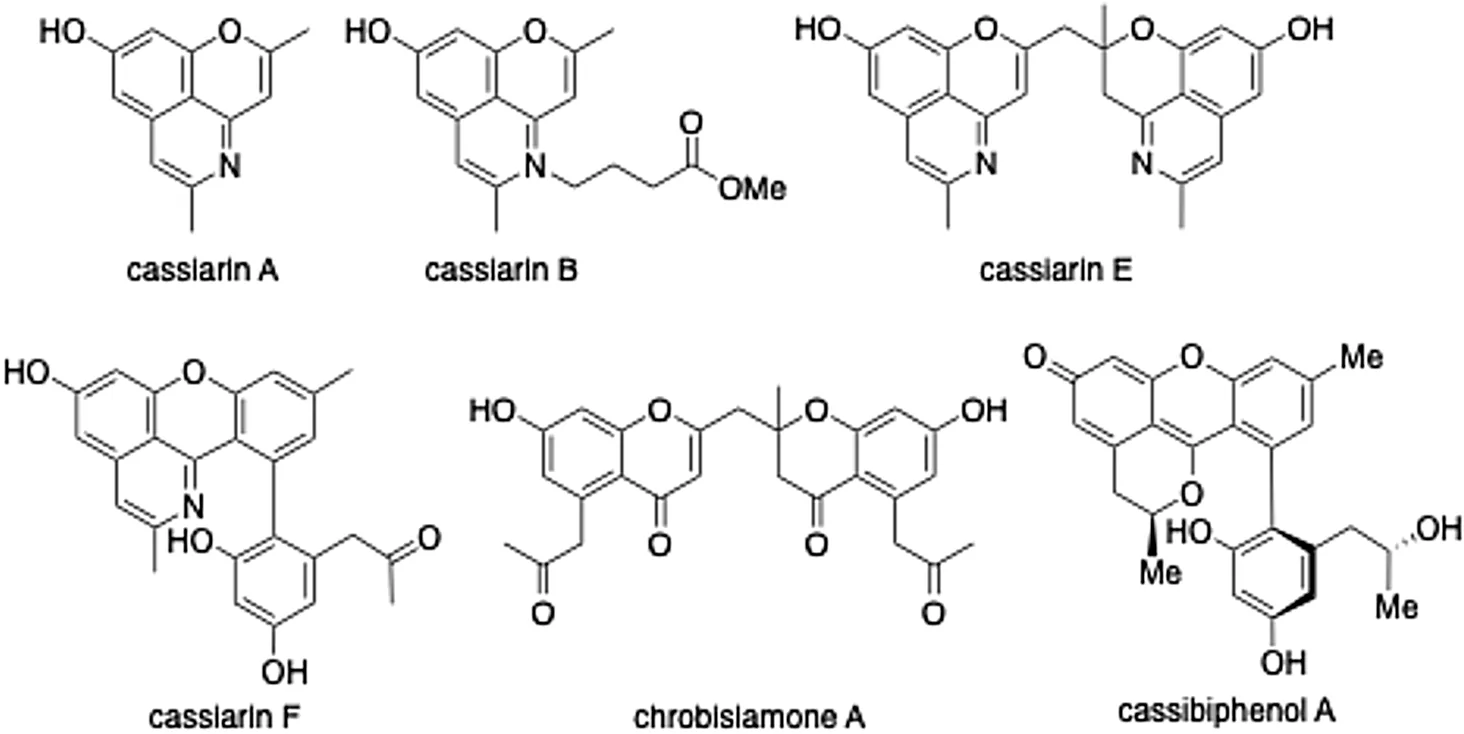
Structures of cassiarins and their related alkaloids

In addition, cassiarin F was identified as a novel tetracyclic alkaloid featuring a biaryl moiety, representing a further level of structural complexity within this family [49]. Collectively, these findings demonstrate that the cassiarin framework is capable of extensive structural diversification, including monomeric, dimeric, and higher-order polycyclic architectures (see Fig. 8).

**Related compounds and biosynthetic consideration**. alongside the cassiarin series, structurally related compounds have been isolated, providing insight into possible biosynthetic relationships. Chrobisiamone A, a bischromone dimer, represents a plausible precursor in the biosynthetic pathway leading to cassiarin-type alkaloids [45, 51]. In addition, cassibiphenols A and B, tetracyclic compounds with biphenyl-like frameworks, further illustrate the structural diversity accessible from chromone-derived intermediates [52].

Biomimetic studies have supported these biosynthetic hypotheses. For example, cyclization of 5-acetonyl-7-hydroxy-2-methylchromone has been shown to generate cassiarin A under appropriate conditions, providing experimental evidence for a plausible biosynthetic pathway. These studies highlight the importance of simple chromone precursors in generating structurally complex alkaloids through intramolecular and intermolecular transformations.

**Total synthesis and structure–activity relationships**. The unique structural features and biological activities of cassiarins have stimulated extensive synthetic studies. Efficient synthetic routes to cassiarin A have been established, and total synthesis of more complex analogues such as cassiarin F has been achieved using key transformations including Suzuki coupling and intramolecular cyclization [49].

**Fig. 9:**
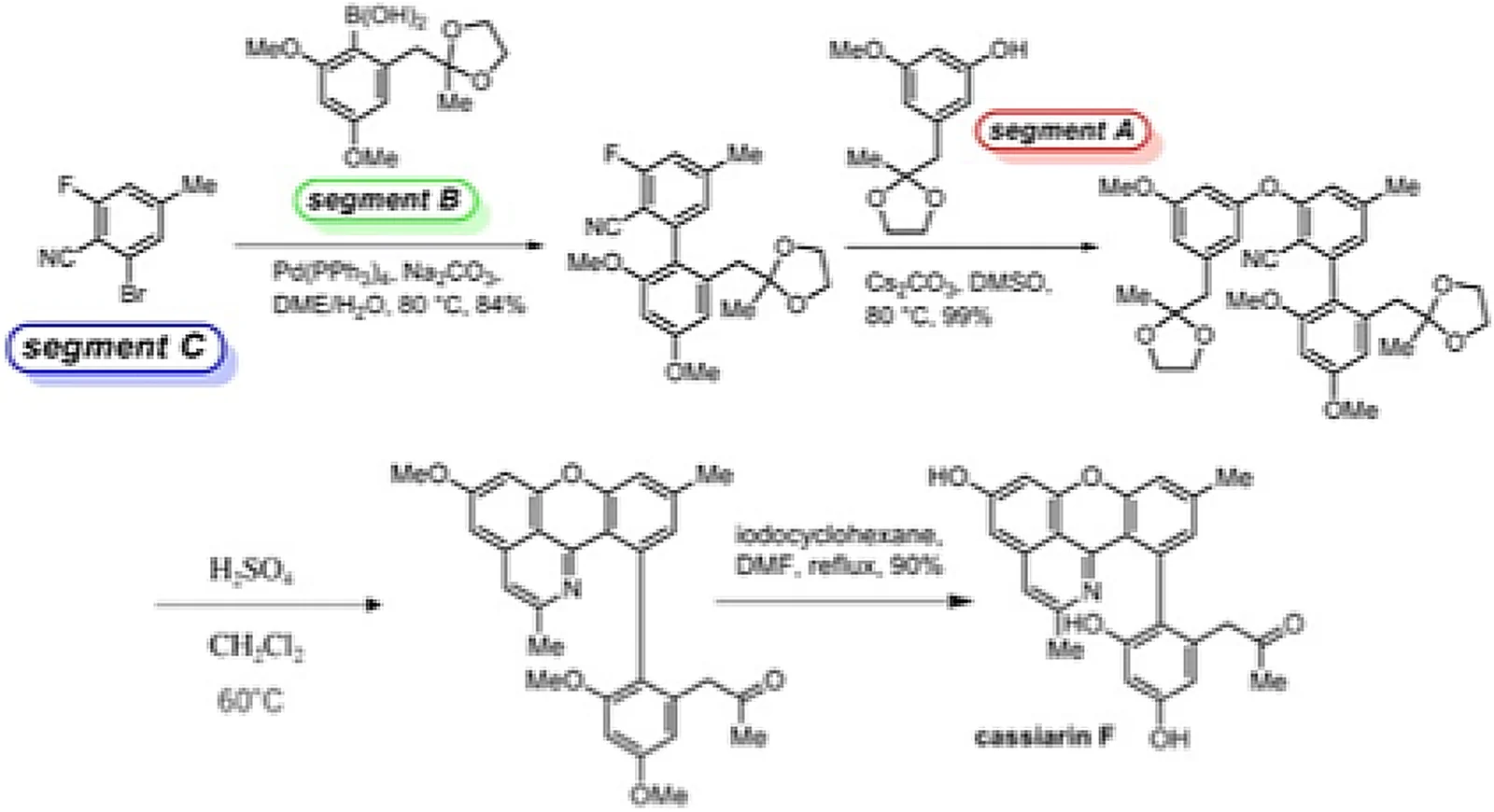
Total synthesis of cassiarin F

Synthetic efforts have also enabled the preparation of a variety of derivatives, including esterified, etherified, and nitrogen-substituted analogues. Evaluation of these compounds revealed important structure–activity relationships. In particular, the presence of both a hydroxyl group and a nitrogen atom in the parent scaffold appears to be critical for maintaining potent antiplasmodial activity, while bulky substituents tend to reduce activity. These findings demonstrate that relatively small structural modifications can significantly influence biological activity (see Fig. 9).

**Vasorelaxant activity and its pharmacological implications**. In addition to their antimalarial activity, cassiarins have been shown to exhibit vasorelaxant effects. Cassiarin A induces concentration-dependent relaxation in rat vascular preparations, and this effect is largely mediated by endothelium-derived nitric oxide (NO). The involvement of NO signaling and potassium channels suggests that cassiarin A modulates vascular tone through both endothelial and smooth muscle mechanisms [48].

This dual activity is of particular interest in the context of malaria pathophysiology. Severe malaria is associated with cytoadherence of parasitized red blood cells to vascular endothelium, leading to impaired microcirculation. Agents that enhance NO production and improve endothelial function may reduce this cytoadherence. Therefore, the vasorelaxant activity of cassiarin A may complement its direct antiparasitic effect, providing a multifaceted approach to malaria treatment.

**Fig. 10:**
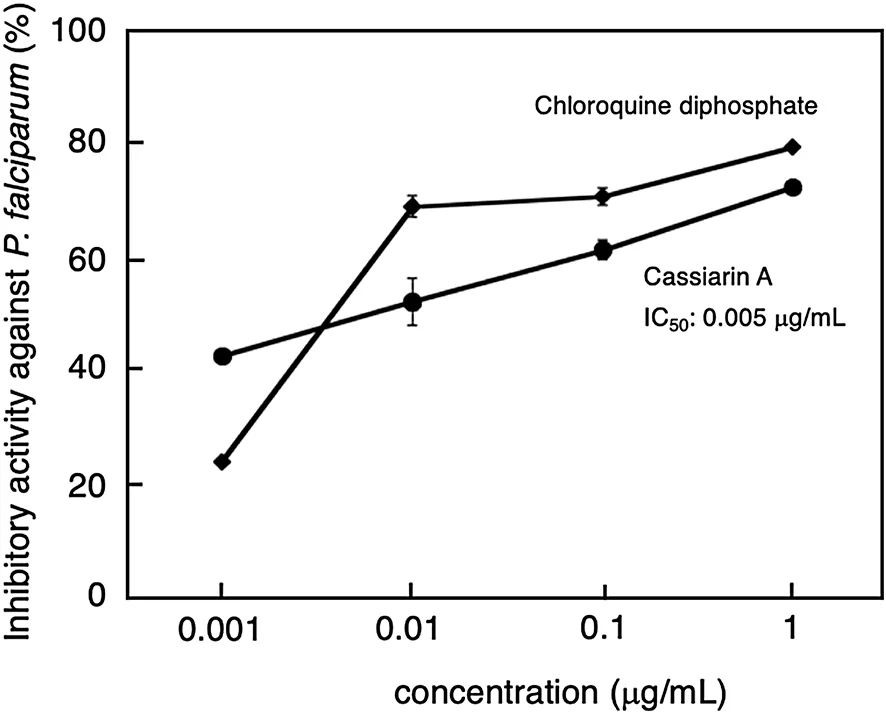
Antimalarial activity of cassiarin A against *P. falciparum* 3D7. The antimalarial activities of the extract and isolated compounds were determined by the procedure described by Budimulya et al*.* Each compound was separately dissolved in DMSO (10 mM). The malarial parasite *P. falciparum* was propagated in a 24-well culture plate in the presence of a wide range of concentrations of each compound. The growth of the parasite was monitored by making a blood smear fixed with MeOH and stained with Giemsa stain. Cassiarin A showed potent antiplasmodial activity (IC_50_: 0.005 mg/mL) comparable to that (IC_50_: 0.006 mg/mL) of chloroquine diphosphate against *P. falciparum* strain 3D7 (chloroquine-sensitive clone)

**Convergence to antimalarial activity**. Despite the structural diversity and multiple biological activities observed within this series, the research ultimately converges on antimalarial activity as the most significant pharmacological outcome. Cassiarin A exhibits potent in vitro activity comparable to chloroquine and demonstrates in vivo efficacy, supporting its potential as a lead compound. Other cassiarin analogues and related compounds also show varying degrees of antiplasmodial activity, indicating that this chemical class constitutes a valuable source of antimalarial agents [46] (see Fig. 10).

## Autophagy-inducing bisindole alkaloids from *Leuconotis* species

Plants of the family Apocynaceae are well known as rich sources of monoterpene indole alkaloids, including monomeric, dimeric, and more complex oligomeric structures. In our studies on tropical Apocynaceae plants, particular attention has been paid to *Leuconotis* species, especially *Leuconotis griffithii* and *Leuconotis eugeniifolia*, which have yielded structurally diverse indole and bisindole alkaloids.

**Fig. 11:**
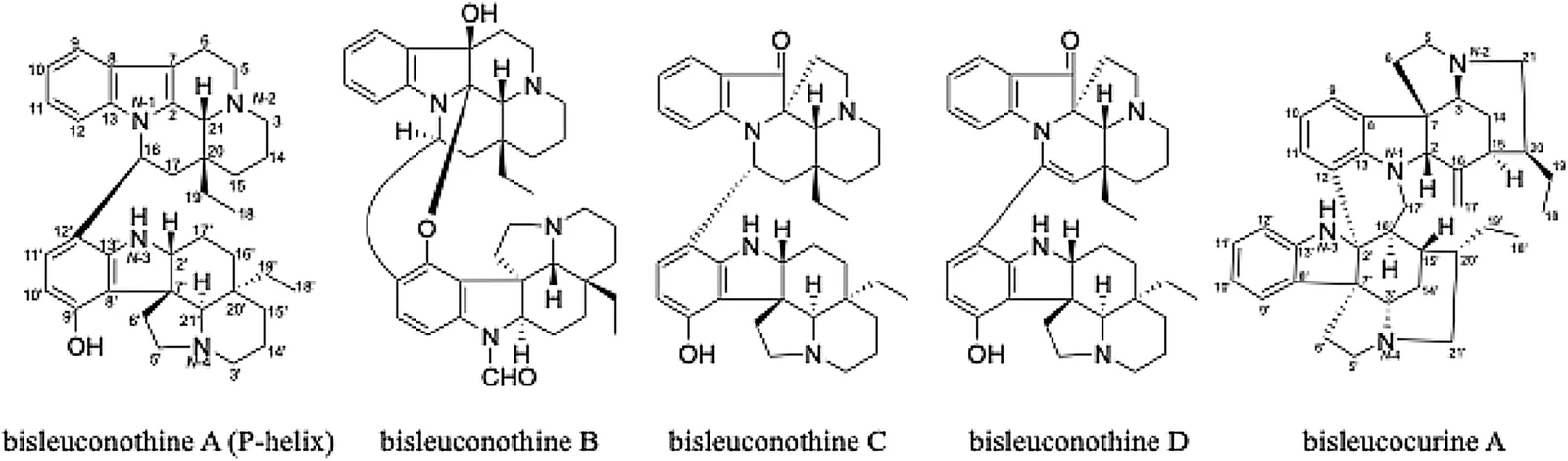
Structures of bisleuconothines A-D and bisleucocurine A from *Leuconotis griffithii*

Initial phytochemical investigations of *L. griffithii* led to the isolation of several structurally unique alkaloids. From the leaves, bisleucocurine A was isolated as a novel bisindole alkaloid consisting of two strychnan-type skeletons, and it exhibited cytotoxicity against several human cancer cell lines [53]. In addition, leucoridine A *N*-oxide and related indole alkaloids were obtained from the same plant, and their antiplasmodial activities against *Plasmodium falciparum* 3D7 were evaluated. These findings indicated that *L. griffithii* is a productive source of both structurally unusual and biologically active indole alkaloids [54] (see Fig. 11).

**Fig. 12:**
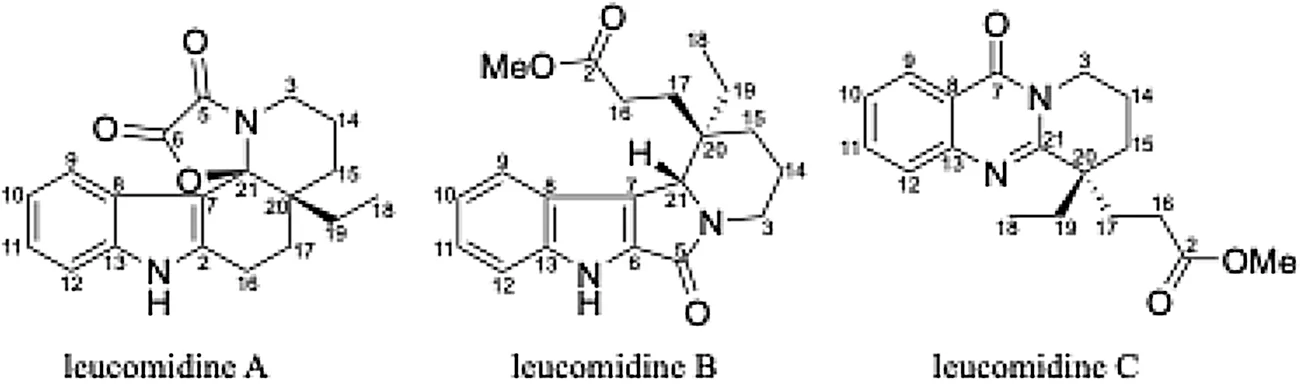
Structures of leucomidines A-C from *Leuconotis griffithii*

Further studies on the bark of *L. griffithii* resulted in the isolation of bisleuconothine A, a bisindole alkaloid composed of eburnane and aspidosperma units [55]. Its structure, including absolute configuration, was elucidated by 2D NMR analysis and X-ray crystallography, and the compound showed cell growth inhibitory activity against several human cancer cell lines. Subsequent isolation work afforded modified analogues, bisleuconothines B–D, which expanded the structural diversity of this eburnane–aspidosperma-type alkaloid class; however, these analogues were noncytotoxic against A549 cells under the tested conditions [56]. In parallel, leucomidines A–C, including indole and quinazoline alkaloids, were also isolated from *L. griffithii*, further supporting the biosynthetic versatility of this species [57] (see Fig. 12).

**Fig. 13:**
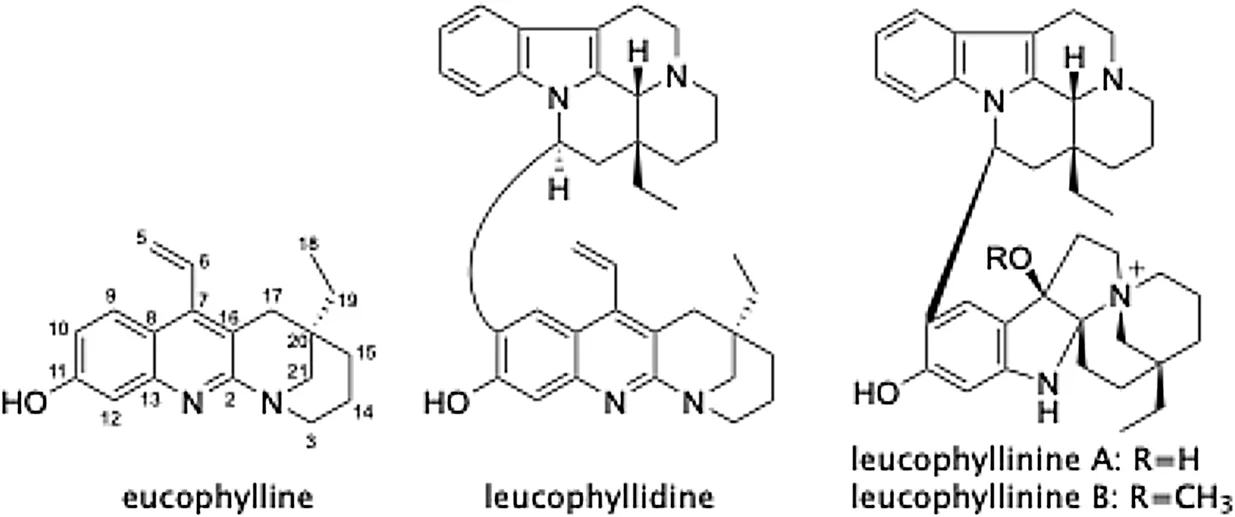
Structures of eucophylline, leucophyllidine, and leucophyllinines A and B from *Leuconotis eugeniifolia*

A second focus within the genus was *L. eugeniifolia*. From this species, eucophylline, a tetracyclic vinylquinoline alkaloid, was isolated together with leucophyllidine [58]. Leucophyllidine showed iNOS inhibitory activity and decreased iNOS protein expression in a dose-dependent manner. Additional studies on *L. eugeniifolia* led to the discovery of leucophyllinines A and B, bisindole alkaloids consisting of eburnane and quebrachamine-type units, which exhibited antiplasmodial activity against *P. falciparum* 3D7 [59]. These results demonstrate that *Leuconotis* species generate a wide range of dimeric indole alkaloids with cytotoxic, anti-inflammatory, and antiplasmodial properties (see Fig. 13).

Among these compounds, bisleuconothine A became the key molecule for mechanistic investigation. Although initially identified through its cytostatic activity, detailed studies revealed that bisleuconothine A did not primarily induce apoptosis. Annexin V/propidium iodide staining and analysis of apoptosis-related proteins showed no marked apoptotic response. Instead, bisleuconothine A increased LC3 lipidation in A549 and MCF7 cells, suggesting induction of autophagy-related processes.

Further mechanistic analysis showed that bisleuconothine A induced autophagosome formation while preventing its degradation. Western blotting and immunofluorescence studies demonstrated increased LC3-II accumulation, consistent with autophagosome formation. At the signaling level, bisleuconothine A downregulated phosphorylation of AKT, PRAS40, and p70S6K, indicating inhibition of the AKT–mTOR pathway. Since mTOR functions as a major negative regulator of autophagy, suppression of this pathway provides a plausible mechanism for bisleuconothine A-induced autophagosome formation [60].

**Fig. 14:**
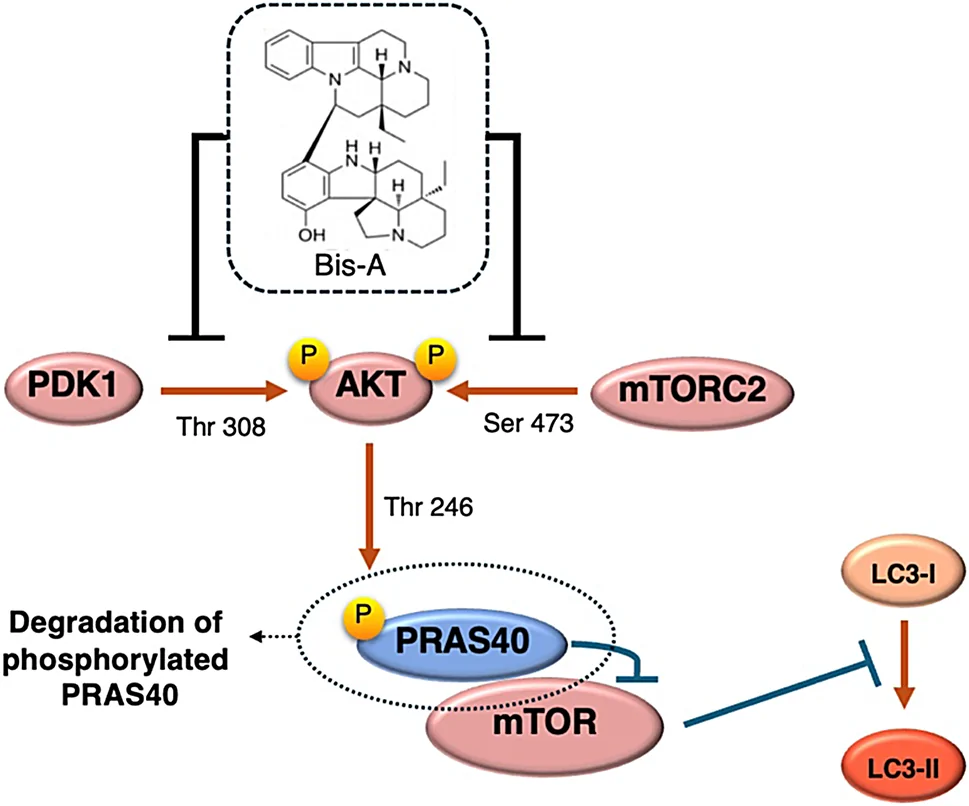
Mode of action for LC3 lipidation by bisleuconothine A. The mechanism underlying the growth-inhibitory effect of bisleuconothine A (Bis-A) on A549 cells was investigated. Analysis using Annexin V and propidium iodide (PI) staining revealed that Bis-A did not induce apoptosis. Furthermore, the expression levels of all apoptosis-related proteins examined remained unaffected by Bis-A treatment. It was found that Bis-A enhanced LC3 lipidation in both A549 and MCF7 cells, suggesting that the growth-inhibitory effect of Bis-A might be attributed to the induction of autophagy. Subsequent investigations using Western blotting and immunofluorescence staining demonstrated that Bis-A induced autophagosome formation while simultaneously inhibiting autophagosome degradation. Further analysis revealed that Bis-A reduced the phosphorylation of AKT, PRAS40 (a downstream target of AKT and an inhibitor of mTOR), and p70S6K (an mTOR-dependent kinase). These reductions in phosphorylation suggest that the enhanced LC3 lipidation may result from mTOR inactivation. Thus, it is suggested that the growth-inhibitory effect of Bis-A involves the induction of autophagosome formation, driven by interference with the AKT-mTOR signaling pathway

Taken together, the *Leuconotis* studies illustrate a progression from phytochemical exploration of structurally unusual Apocynaceae alkaloids to detailed biological mechanism analysis. Although many compounds from *Leuconotis* species exhibit cytotoxic or antiplasmodial activities, bisleuconothine A is particularly notable because its cytostatic effect is associated with autophagosome formation through interference with AKT–mTOR signaling. This case therefore represents an example in which screening of tropical plant alkaloids led not only to the discovery of new chemical scaffolds but also to the identification of a natural product modulating autophagy-related signaling (see Fig. 14).

## Cyclic peptides from flaxseed: inhibition of osteoclast differentiation by cyclolinopeptide F

**Fig. 15:**
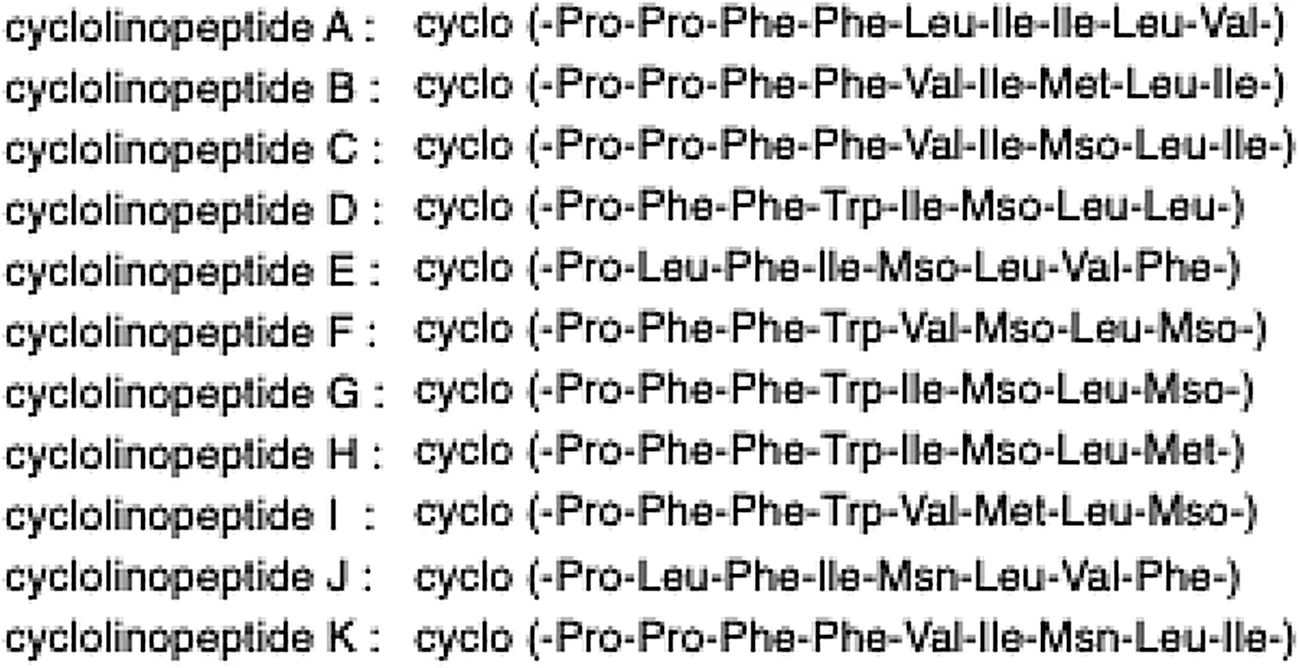
Structures of cyclolinopeptides from flaxseed (*Linum usitatissimum*)

Cyclic peptides represent a structurally distinct class of natural products widely distributed in higher plants, often exhibiting unique conformational rigidity, enhanced lipophilicity, and diverse biological activities [61]. Among these, flaxseed (*Linum usitatissimum*, Linaceae) is a particularly rich source of cyclic peptides known as cyclolinopeptides, which have attracted attention due to their structural diversity and pharmacological potential [62–65] (see Fig. 15).

**Discovery and structural features of cyclolinopeptides**. Phytochemical investigations of flaxseed have led to the identification of numerous cyclolinopeptides, typically composed of 8–10 amino acid residues arranged in a macrocyclic structure. These peptides often differ subtly in amino acid composition and oxidation state, particularly at methionine residues, giving rise to a series of structurally related analogues [62–64]. The conformational restriction imposed by cyclization is considered to contribute to their biological activity by enhancing target affinity and stability [65].

Cyclolinopeptides have been reported to exhibit a range of biological activities, including immunosuppressive and antimalarial effects [62]. However, their role in bone metabolism remained largely unexplored until systematic biological screening was conducted.

**Identification of osteoclast differentiation inhibitory activity**. In our search for inhibitors of osteoclast differentiation, cyclolinopeptides isolated from flaxseed were evaluated using TRAP activity as an indicator of osteoclastogenesis. As a result, several cyclolinopeptides were found to inhibit osteoclast differentiation in a dose-dependent manner without affecting cell viability [66].

Among them, cyclolinopeptides F, H, and I exhibited particularly potent activity, surpassing that of known reference compounds such as curcumin in the same assay system. These findings revealed, for the first time, that flaxseed-derived cyclic peptides can function as modulators of osteoclast differentiation.

**Focus on cyclolinopeptide F**. Cyclolinopeptide F (CL-F) was selected as a representative compound for further mechanistic investigation due to its relatively high abundance in flaxseed and its potent inhibitory activity. CL-F inhibited RANKL-induced osteoclastogenesis in a dose-dependent manner (IC₅₀ ≈ 0.58 μM) without cytotoxic effects, indicating that its activity is not due to nonspecific cell damage.

Detailed analysis of the differentiation process revealed that CL-F primarily acts at the early stage of osteoclastogenesis, particularly during the macrophage colony-stimulating factor (M-CSF)-induced transition from myeloid precursor cells to monocyte/macrophage lineage cells. In addition, CL-F showed a weaker but measurable inhibitory effect on the later differentiation stage induced by RANKL [67, 68].

**Mechanistic insight: c-Fos/RANK signaling axis**. At the molecular level, CL-F was found to downregulate the expression of c-Fos, a key transcription factor involved in osteoclast differentiation. Since c-Fos is required for the induction of receptor activator of nuclear factor-κB (RANK), its suppression leads to decreased RANK expression in osteoclast precursor cells.

Consistent with this, CL-F treatment significantly reduced RANK expression in M-CSF-induced monocyte/macrophage lineage cells, thereby attenuating downstream RANKL signaling. Given that RANK–RANKL interaction is essential for the activation of NFATc1, the master regulator of osteoclast differentiation, inhibition of the c-Fos/RANK axis effectively suppresses the osteoclastogenic transcriptional program.

This mechanism highlights an early-stage regulatory effect distinct from compounds that directly inhibit osteoclast function at later stages. **Pharmacological implications and related activities**. In addition to osteoclast differentiation inhibition, cyclolinopeptides have also been reported to exhibit antitumor activity in giant-cell tumor of bone (GCTB) models, where suppression of RANKL signaling was associated with reduced cell proliferation and altered cell cycle progression. These findings further support the role of cyclolinopeptides as modulators of RANK/RANKL signaling pathways [67, 68].

**Fig. 16:**
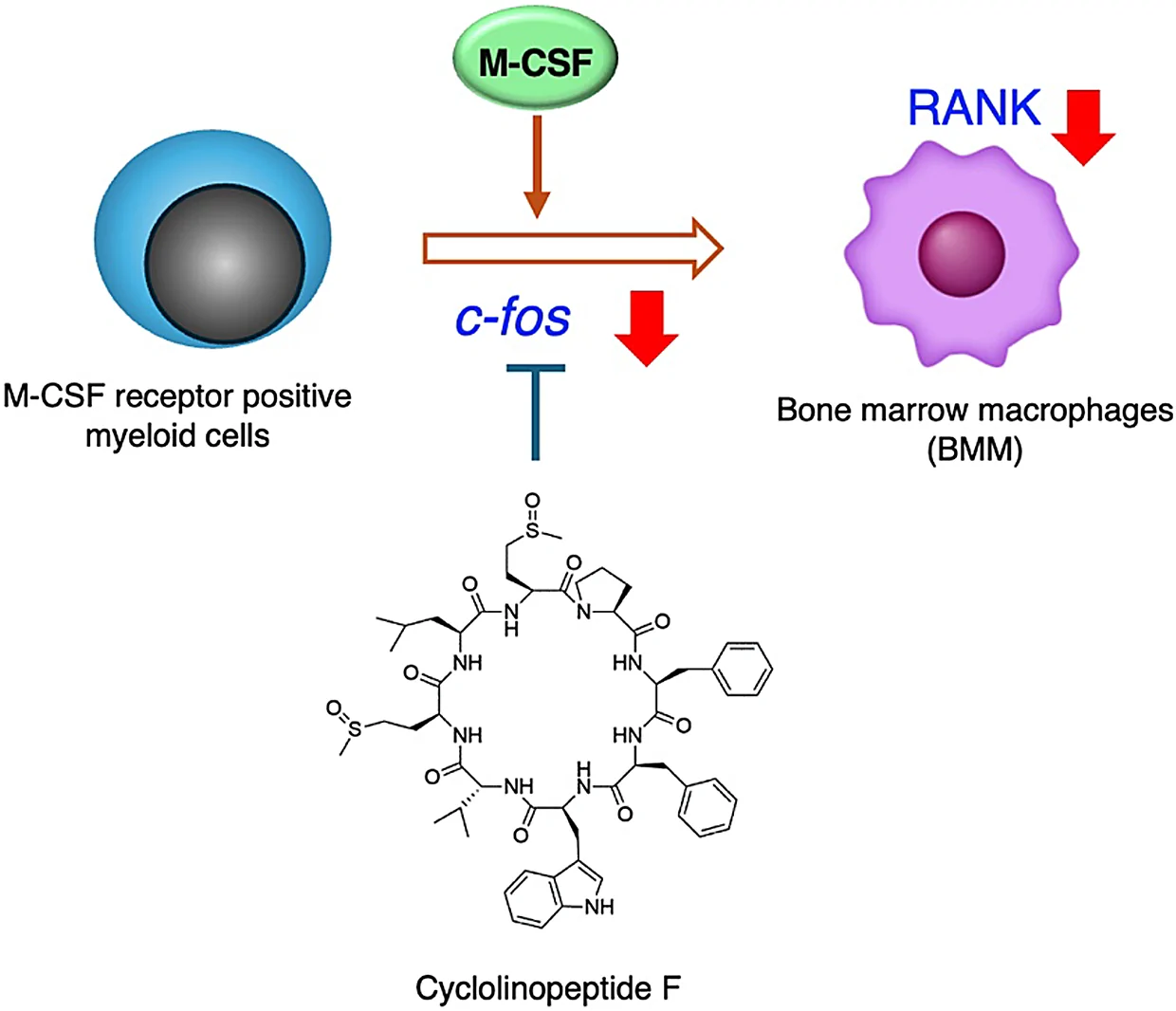
Proposed mode of action for cyclolinopeptide F from flaxseed. The inhibitory effects of cyclolinopeptide F (CL-F)—a representative cyclolinopeptide (CL)—on osteoclast differentiation were investigated in detail. CL-F inhibited RANKL-induced osteoclast differentiation in a dose-dependent manner (IC_50_: 0.58 µM) without exhibiting cytotoxicity. This inhibitory effect was primarily observed during the M-CSF (macrophage colony-stimulating factor)-induced proliferation and differentiation stage, in which immature myeloid cells transition into the monocyte/macrophage (M/Mϕ) lineage. Additionally, CL-F slightly inhibited the RANKL-induced differentiation stage from M/Mϕs to mature osteoclasts. CL-F treatment reduced the expression of the RANKL receptor (RANK) in M-CSF-induced M/Mϕs (*i.e*., osteoclast precursors). c-Fos is known to be essential for RANK expression in osteoclast precursors derived from myeloid cells via M-CSF induction. Therefore, it is suggested that CL-F inhibits osteoclast differentiation by suppressing *c-fos* expression, thereby leading to reduced RANK expression in M-CSF-induced osteoclast precursors

The dual activity of cyclolinopeptides in both osteoclastogenesis and tumor-related processes suggests that these compounds may have broader therapeutic potential in diseases involving dysregulated bone remodeling and associated pathological conditions (see Fig. 16).

## Tubulin polymerization inhibitory peptides from *Celosia argentea*

Cyclic peptides from higher plants represent a unique class of natural products with structurally constrained frameworks and diverse biological activities [61]. Among these, peptides isolated from *Celosia argentea* (Amaranthaceae) have attracted considerable attention due to their unusual bicyclic architectures and potent antimitotic activity [69–73].

**Discovery of bicyclic peptides from Celosia argentea.** Phytochemical investigation of the seeds of *C. argentea*, a traditional medicinal plant used in East Asia, led to the isolation of a series of structurally unique bicyclic peptides, including celogentins A–C [70, 71], together with the known peptide moroidin [69]. These compounds possess highly unusual structural features, notably a bicyclic ring system formed through nonstandard linkages involving β-substituted leucine (βs-Leu), tryptophan, and histidine residues.

**Fig. 17:**
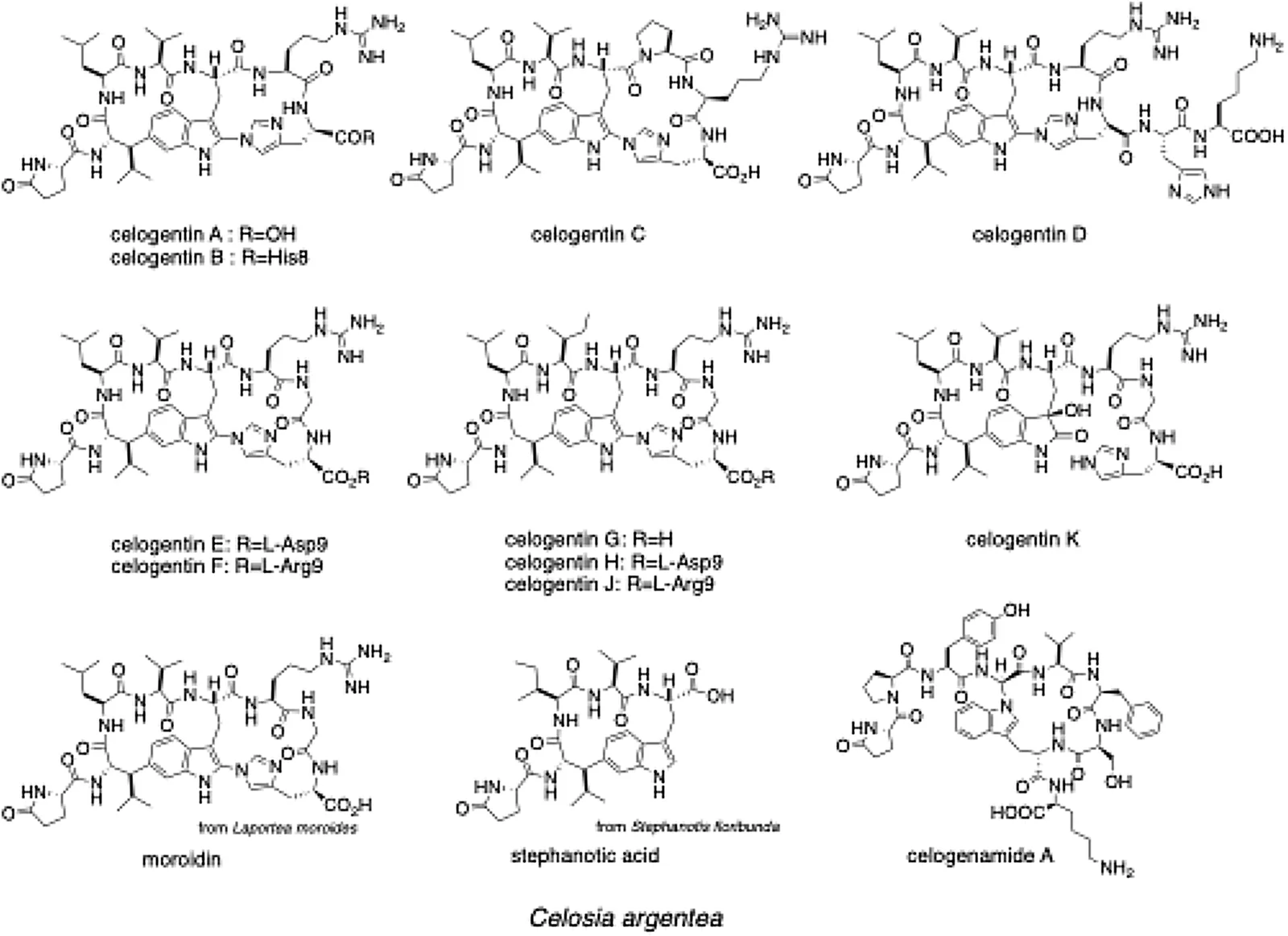
Structures of celogentins and their related cyclic peptides from *Celosia argentea*

Subsequent studies expanded this family to include celogentins A–H and J [71], as well as related cyclic peptides such as celogentin K [72] and celogenamide A [73], thereby revealing remarkable structural diversity within this class. Detailed structural elucidation was achieved by extensive spectroscopic analyses, including multidimensional NMR and mass spectrometry, as well as X-ray crystallography in selected cases (see Fig. 17).

**Structural characteristics and diversity.** The celogentins are characterized by a rigid bicyclic scaffold incorporating unusual C–C and C–N linkages between amino acid residues. In particular, the connection between the β-carbon of βs-Leu and the indole ring of tryptophan represents a distinctive structural motif critical for biological activity.

In contrast, celogentin K possesses a modified structure featuring a 3-hydroxyoxindole moiety derived from oxidative modification of the tryptophan residue, resulting in a monocyclic framework distinct from the bicyclic celogentins [72]. Similarly, celogenamide A represents a structurally different cyclic peptide lacking the characteristic bicyclic connectivity [73].

These structural variations provide a valuable framework for understanding the relationship between peptide conformation and biological activity.

**Tubulin polymerization inhibitory activity.** Microtubules, composed of α/β-tubulin heterodimers, play a central role in mitotic spindle formation and cell division. Disruption of tubulin polymerization is therefore a well-established strategy for anticancer drug development.

Celogentins and related peptides were found to inhibit tubulin polymerization in a concentration-dependent manner. Among them, celogentin C exhibited particularly potent activity, being approximately four times more active than moroidin [69], a previously known tubulin polymerization inhibitor. These findings were further supported by studies demonstrating that several celogentins effectively suppress microtubule formation, leading to mitotic arrest of eukaryotic cells.

In contrast, structurally modified analogues such as celogenamide A did not show significant inhibition of tubulin polymerization, indicating that specific structural features are essential for activity.

**Structure–activity relationship**. Structure–activity relationship (SAR) studies revealed that the bicyclic ring system of celogentins is crucial for interaction with tubulin. The unique conformational constraint imposed by the bicyclic framework, together with the presence of unusual amino acid linkages, appears to be essential for effective binding to the tubulin target.

In particular, the size and geometry of the bicyclic ring, as well as the connectivity between βs-Leu, tryptophan, and histidine residues, were found to significantly influence activity. The loss or modification of this structural motif, as observed in monocyclic derivatives, leads to a marked decrease or complete loss of activity.

**Fig. 18:**
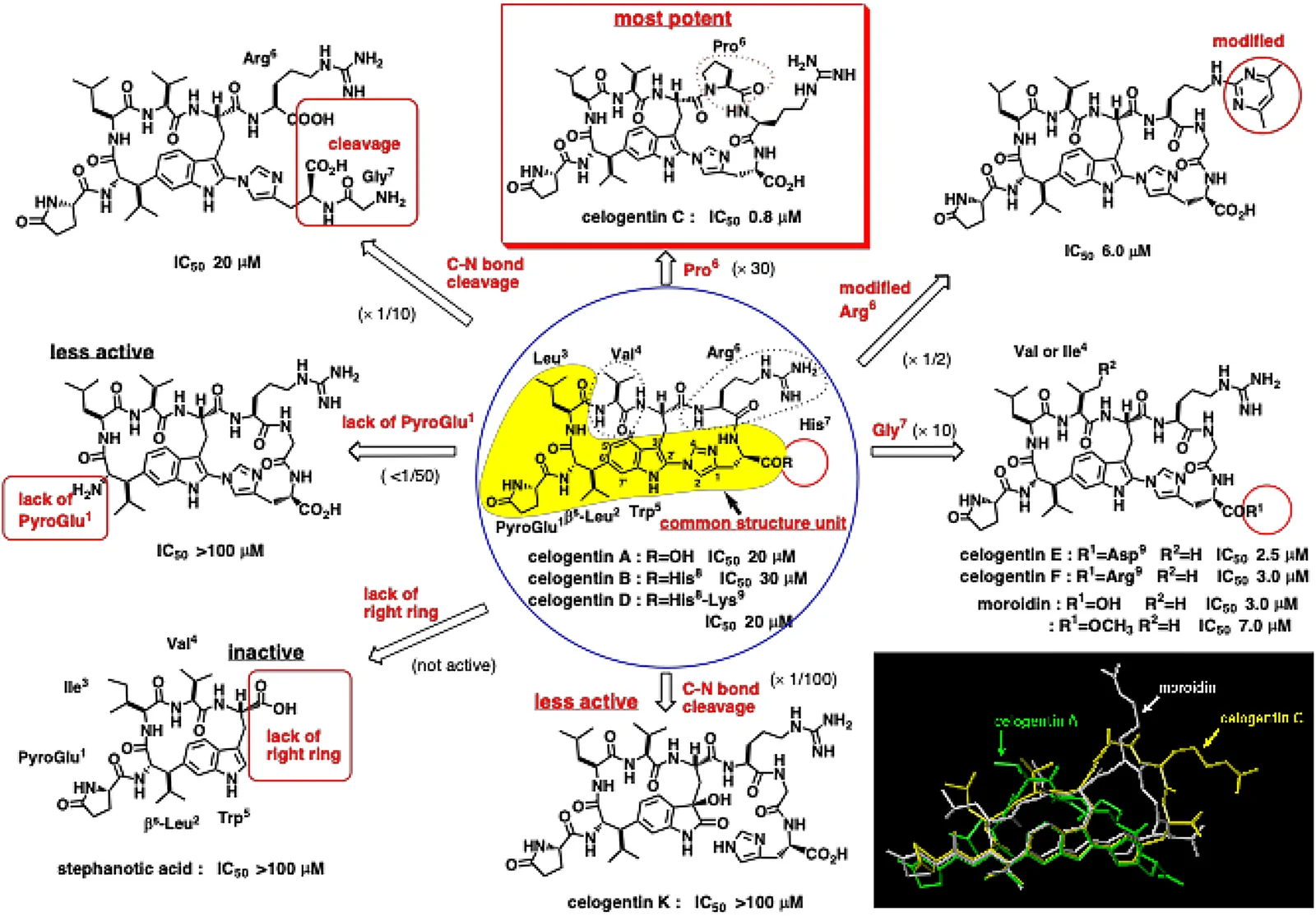
Summary of SAR studies of celogentins and their related peptides

These findings highlight the importance of three-dimensional structural features in determining the biological function of cyclic peptides (see Fig. 18).

**Pharmacological implications**. The potent inhibition of tubulin polymerization by celogentins suggests their potential as lead compounds for the development of antimitotic agents. Their mechanism of action is comparable to that of other natural antimitotic compounds, such as vinca alkaloids and peptide-based inhibitors, which target microtubule dynamics.

Furthermore, the structurally unique bicyclic framework of celogentins represents a novel scaffold for drug discovery, offering opportunities for the design of synthetic analogues with improved pharmacological properties.

## Mollugin and its derivatives: from natural product to mechanism-oriented drug development

**Fig. 19:**
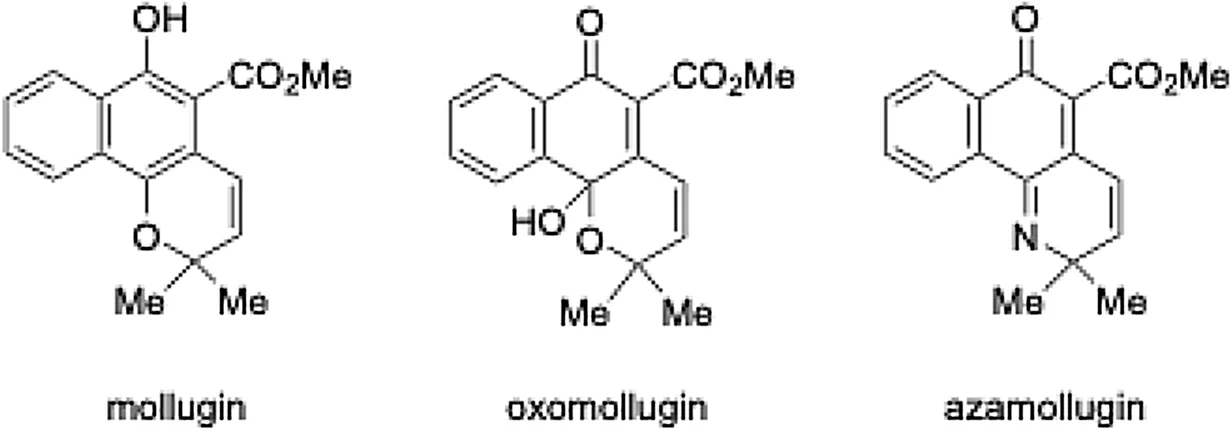
Structures of mollugin from *Rubia cordifolia* and its derivatives, oxomollugin and azamollugin

Mollugin, a naphthoquinone derivative isolated from *Rubia cordifolia* (Rubiaceae), has long been recognized as a bioactive natural product with reported anti-inflammatory, anti-tumor, and neuroprotective effects [74–76]. However, a critical reassessment of its biological activity revealed a pivotal finding: the apparent pharmacological effects attributed to mollugin are largely due to its oxidized derivative, oxomollugin, rather than the parent compound itself [77] (see Fig. 19).

In this foundational study, oxomollugin was identified as a degradation product formed from mollugin under experimental conditions and was shown to be the true active species responsible for inhibiting lipopolysaccharide (LPS)-induced NF-κB activation. Mechanistic investigation demonstrated that oxomollugin suppresses Toll-like receptor 4 (TLR4)-mediated signaling by interfering with the recruitment and activation of essential adaptor proteins, including myeloid differentiation primary response protein 88 (MyD88), interleukin-1 receptor-associated kinase 4 (IRAK4), and tumor necrosis factor receptor-associated factor 6 (TRAF6), thereby preventing downstream signal transduction leading to NF-κB activation [79].

This finding necessitated a fundamental reinterpretation of previously reported activities of mollugin. Although mollugin itself had been widely studied, it became evident that its biological effects may have been influenced by the formation of oxomollugin during isolation, storage, or assay conditions. This case highlights the importance of chemical transformation and stability in the accurate evaluation of natural product bioactivity.

Detailed mechanistic studies further clarified that oxomollugin acts at multiple stages of the TLR4 signaling cascade. Specifically, oxomollugin inhibits the association of MyD88 with IRAK4 at the initial phase of signaling, delays the activation and degradation of IRAK1, and suppresses the ubiquitination of TRAF6. In addition, it prevents the formation of the TRAF6–TAK1/TAB2 complex, which is required for the activation of IκB kinase (IKK). As a consequence, phosphorylation of IKKα/β is reduced, leading to inhibition of IκB degradation and subsequent NF-κB nuclear translocation [79].

Furthermore, oxomollugin also suppresses the expression and modification of IRAK2, a key mediator of the late phase of TLR4 signaling. This dual inhibition of both IRAK1-dependent early signaling and IRAK2-dependent sustained signaling suggests that oxomollugin exerts a comprehensive suppressive effect on TLR4-mediated inflammatory responses. The ability to target both phases of signaling distinguishes oxomollugin from compounds that act on a single step within the pathway [79].

Building on this mechanistic insight, efforts were directed toward the development of structurally modified derivatives with improved activity. Among these, azamollugin, an aza-modified analogue of oxomollugin, was designed and synthesized [78]. Azamollugin exhibited significantly enhanced inhibitory activity on nitric oxide production and demonstrated broader suppression of inflammatory signaling pathways [80].

Importantly, azamollugin was found to inhibit not only the MyD88-dependent pathway leading to NF-κB activation but also the TRIF-dependent pathway responsible for interferon regulatory factor 3 (IRF3) activation and interferon-β (IFN-β) production. Since TLR4 uniquely activates both MyD88- and TRIF-dependent pathways, the dual inhibitory effect of azamollugin represents a significant advantage in modulating complex inflammatory responses.

Structure–activity relationship (SAR) analysis of the mollugin series reveals a clear trend in activity enhancement. The parent compound, mollugin, shows limited or inconsistent activity, whereas oxidation to form oxomollugin results in a marked increase in inhibitory potency. Further modification through nitrogen incorporation, as in azamollugin, leads to additional enhancement of activity and expansion of the inhibitory spectrum. These findings underscore the importance of oxidative modification and heteroatom introduction in optimizing the pharmacological properties of natural product-derived scaffolds.

From a pharmacological perspective, mollugin derivatives represent a class of small-molecule modulators targeting intracellular signaling pathways involved in inflammation. Unlike peptide-based natural products described in previous sections, which primarily interact with structural or enzymatic targets, mollugin derivatives act on signal transduction processes, particularly at early stages of innate immune activation. Their ability to suppress TLR4-mediated signaling at multiple levels suggests potential applications in the treatment of inflammatory and immune-related disorders.

**Fig. 20:**
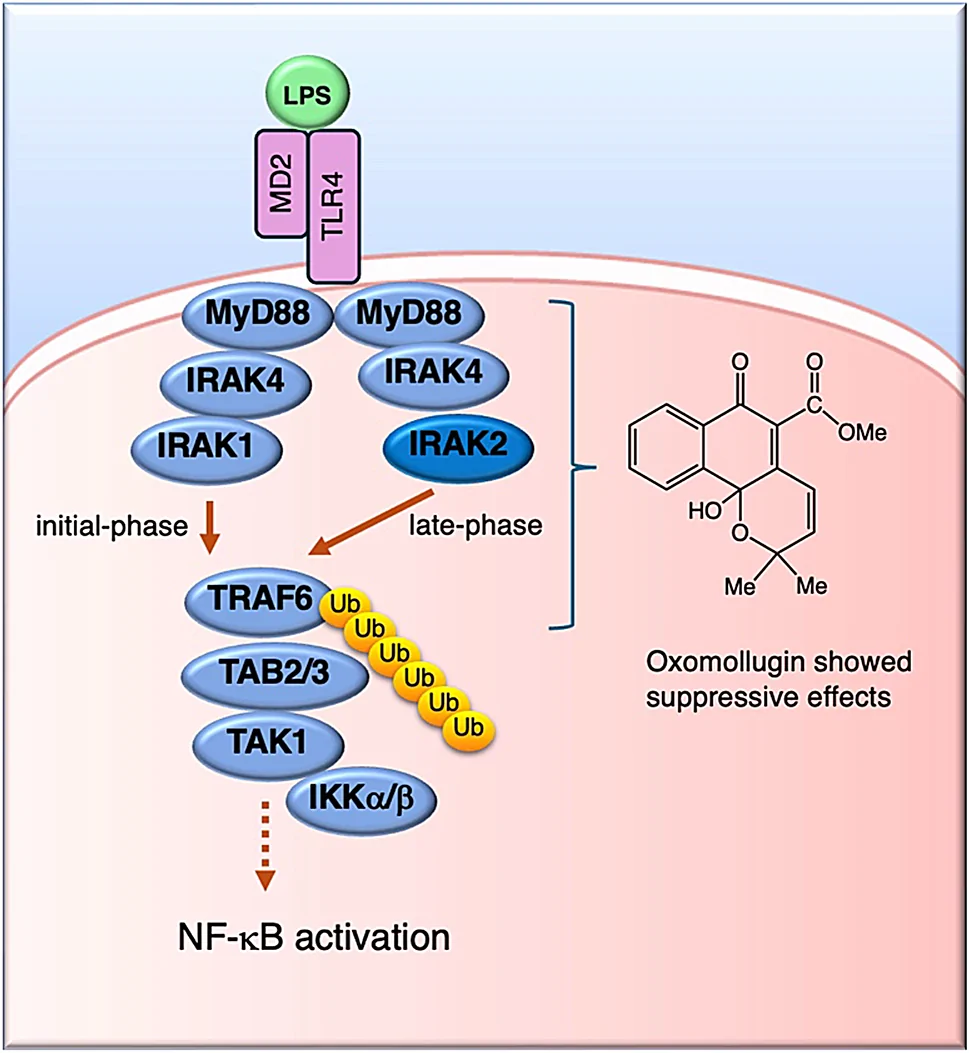
Putative target sites of oxomollugin in TLR4-mediated signals leading to NF-kB activation. The inhibitory activity of oxomollugin—a degradation product of mollugin—was investigated regarding the pathway leading to lipopolysaccharide (LPS)-induced NF-κB activation. Oxomollugin inhibited the LPS-induced association of MyD88, IRAK4, and TRAF6, which are essential factors for the initial activation of TLR4 signaling. Furthermore, oxomollugin suppressed the LPS-induced modification of IRAK1, IRAK2, and TRAF6; the LPS-induced association of TRAF6 with TAK1/TAB2; and the subsequent phosphorylation of IKKα/β. These results suggest that the inhibitory effect of oxomollugin on TLR4 signaling occurs in the vicinity of IRAKs

**Fig. 21:**
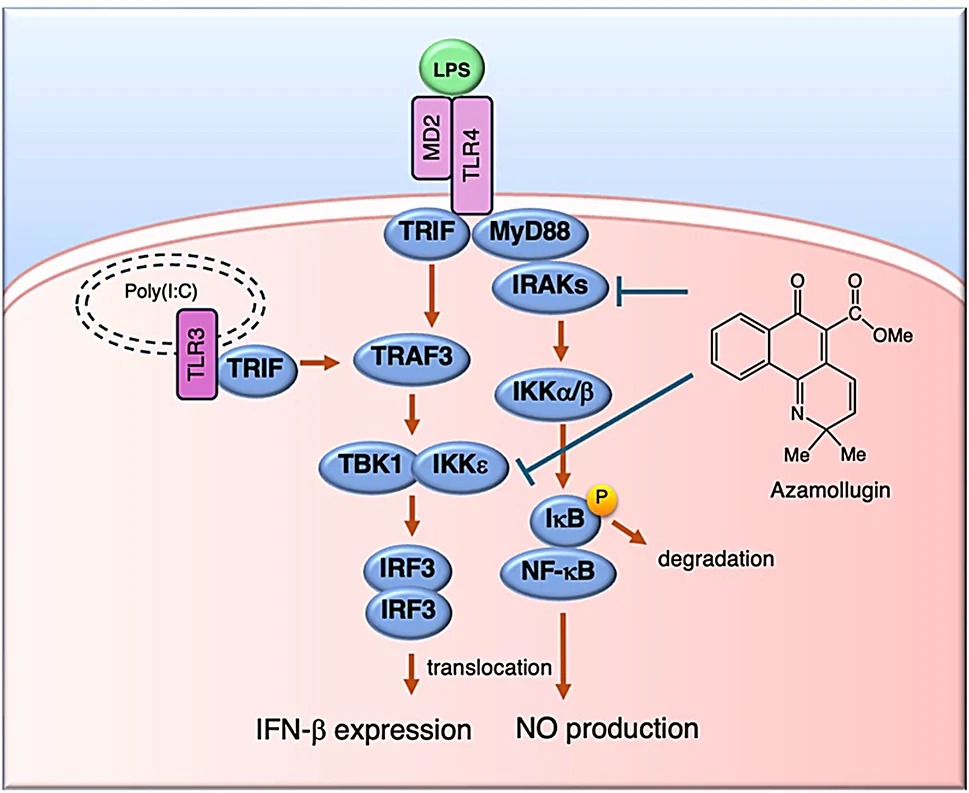
Summary of azamollugin effects in LSP-induced activation signals. It has been revealed that azamollugin not only suppresses the expression of the iNOS gene—which is regulated by NF-κB—but also inhibits LPS-induced IFN-β expression, which is regulated by IRF3. Azamollugin demonstrated inhibitory activity against LPS-induced IRAK1 activation, suggesting an inhibitory effect on the MyD88-dependent pathway. Furthermore, because azamollugin inhibited the LPS-induced phosphorylation of IRF3 and its upstream factors (TBK1 and IKKε), it is suggested to suppress the TLR4-mediated TRIF-dependent pathway. In addition, azamollugin suppressed the phosphorylation of TBK1 and IRF3 induced by poly(I:C), suggesting an inhibitory effect on the TLR3-mediated TRIF-dependent pathway. Therefore, it is suggested that azamollugin possesses inhibitory activity against both the MyD88-dependent and TRIF-dependent pathways

In summary, the study of mollugin and its derivatives exemplifies a complete trajectory from natural product identification to mechanism-oriented drug development. The recognition of oxomollugin as the true active species provided a critical foundation for subsequent mechanistic studies and rational derivative design [79]. The development of azamollugin further demonstrates how structural modification can enhance potency and broaden biological activity [80]. This case highlights the importance of integrating chemical transformation, mechanistic analysis, and synthetic design in the discovery and optimization of natural product-based therapeutic leads (see Figs. 20, 21).

## Conclusion

The present review summarizes our studies on the exploration of bioactive natural products from tropical plant genetic resources, highlighting an integrated research strategy that combines biodiversity-driven sampling, bioactivity-guided isolation, structural elucidation, and mechanistic analysis.

Extensive field studies conducted in Southeast Asia enabled the collection and screening of a large number of plant extracts, providing a broad chemical space for natural product discovery. From this systematic exploration, a diverse array of structurally unique compounds was identified, including limonoids, alkaloids, cyclic peptides, and naphthoquinones. Rather than providing an exhaustive catalog, this review focused on seven representative case studies that illustrate the potential of tropical plant-derived natural products as functional seeds for drug discovery.

These case studies demonstrate several key features of natural product-based research. First, tropical plant resources yield compounds with remarkable structural diversity, such as ceramicines (limonoids), cassiarins (alkaloids), bisleuconothine A (bisindole alkaloid) from *Leuconotis griffithii*, and celogentins and cyclolinopeptides (bicyclic peptides) from *Celosia argentea*. Second, these compounds exhibit a wide range of biological activities, including modulation of lipid metabolism, vascular relaxation, antimalarial effects, induction of autophagy, inhibition of osteoclast differentiation, disruption of microtubule dynamics, and suppression of inflammatory signaling.

Importantly, detailed mechanistic studies revealed that many of these natural products act on well-defined molecular targets and signaling pathways. Examples include regulation of the AKT–FoxO1–PPARγ axis by ceramicines, PI3K–cAMP–PKA signaling by calofolic acids, dual antiparasitic and vasorelaxant effects of cassiarins, AKT–mTOR-mediated autophagy induction by bisleuconothine A, suppression of the c-Fos/RANK pathway by cyclolinopeptide F, inhibition of tubulin polymerization by celogentins, and TLR4 signaling modulation by mollugin derivatives.

A notable feature emerging from these studies is the convergence of diverse chemical scaffolds toward biologically relevant mechanisms. While initial screenings often reveal multiple activities, deeper investigation typically identifies a dominant pharmacological function supported by a defined molecular mechanism. This transition from phenotypic observation to mechanistic understanding is essential for advancing natural products toward drug discovery.

Furthermore, several cases demonstrate the importance of chemical transformation and synthetic modification. The identification of oxomollugin as the true active species and the development of azamollugin highlight how subtle structural changes can significantly enhance biological activity and broaden target profiles. Similarly, synthetic studies of cassiarins and SAR analysis of limonoids and peptides provide insights into the structural requirements for activity.

Taken together, these findings underscore the value of tropical plant genetic resources as a rich source of chemically diverse and biologically relevant natural products. The integration of field-based exploration with modern chemical and biological approaches enables the identification of functionally significant molecules that serve as seeds for drug discovery.

Future studies should focus on further elucidation of molecular targets, in vivo validation, and optimization of pharmacokinetic properties. In this context, sustainable access to biodiversity and continued international collaboration will remain essential for advancing natural product research. The strategy described in this review provides a framework for translating biodiversity into mechanistically defined bioactive compounds with therapeutic potential.
